# Protein interactions with metallothionein-3 promote vectorial active transport in human proximal tubular cells

**DOI:** 10.1371/journal.pone.0267599

**Published:** 2022-05-03

**Authors:** Gazal Kalyan, Andrea Slusser-Nore, Jane R. Dunlevy, Chandra S. Bathula, John B. Shabb, Wallace Muhonen, Seema Somji, Donald A. Sens, Scott H. Garrett

**Affiliations:** 1 Department of Pathology, School of Medicine and Health Sciences, University of North Dakota, Grand Forks, ND, United States of America; 2 Department of Biomedical Sciences, School of Medicine and Health Sciences, University of North Dakota, Grand Forks, ND, United States of America; Anatomy, SWITZERLAND

## Abstract

Metallothionein 3 (MT-3) is a small, cysteine-rich protein that binds to essential metals required for homeostasis, as well as to heavy metals that have the potential to exert toxic effects on cells. MT-3 is expressed by epithelial cells of the human kidney, including the cells of the proximal tubule. Our laboratory has previously shown that mortal cultures of human proximal tubular (HPT) cells express MT-3 and form domes in the cell monolayer, a morphological feature indicative of vectorial active transport, an essential function of the proximal tubule. However, an immortalized proximal tubular cell line HK-2 lacks the expression of MT-3 and fails to form domes in the monolayer. Transfection of HK-2 cells with the MT-3 gene restores dome formation in these cells suggesting that MT-3 is required for vectorial active transport. In order to determine how MT-3 imparts this essential feature to the proximal tubule, we sought to identify proteins that interact either directly or indirectly with MT-3. Using a combination of pulldowns, co-immunoprecipitations, and mass spectrometry analysis, putative protein interactants were identified and subsequently confirmed by Western analysis and confocal microscopy, following which proteins with direct physical interactions were investigated through molecular docking. Our data shows that MT-3 interacts with myosin-9, aldolase A, enolase 1, β-actin, and tropomyosin 3 and that these interactions are maximized at the periphery of the apical membrane of doming proximal tubule cells. Together these observations reveal that MT-3 interacts with proteins involved in cytoskeletal organization and energy metabolism, and these interactions at the apical membrane support vectorial active transport and cell differentiation in proximal tubule cultures.

## Introduction

The metallothioneins (MT) are a family of intracellular metal-binding proteins of low molecular weight (6 kD) with a very high number of conserved cysteine residues that allow the efficient binding of transition metals [1]. In both mice and humans, the MT gene family is divided into 4 isoforms based on small differences in sequence and charge, designated as MT-1 through MT-4 [2–4]. The MT-1 and MT-2 family members have been extensively studied due to their role in the homeostasis of zinc (Zn^2+^) and copper (Cu^2+^), redox biology and in the detoxification of heavy metals such as cadmium (Cd^2+^) and mercury (Hg^2+^) [5–7]. In addition, their induced expression upon heavy metal exposure is ubiquitous in almost all tissues, making the MT-1 and MT-2 genes an early model for the study of metal-induced gene regulation [8, 9]. The MT-1 and MT-2 family members are central to the cell’s armamentarium against stress [7, 10]. In contrast, the MT-3 isoform has seen a far less extensive study but possesses several unique features not shared by the MT-1 and MT-2 isoforms. The MT-3 isoform was initially cloned as a human growth inhibitory factor due to its down-regulation in Alzheimer’s disease [11]. Concurrently, this growth inhibitory factor was identified as a metallothionein-like protein [12] and as MT-III, a brain-specific member of the metallothionein gene family [13]. The MT-3 isoform is structurally unique in that it possesses 7 additional amino acids that are not present in any other member of the MT gene family, a 6 amino acid C-terminal sequence, and a Thr in the N-terminal region [11–13]. The unique N-terminal region is required for its neuronal growth inhibitory activity, an activity not shared by other members of the MT gene family [14]. The MT-3 isoform is not induced by heavy metals and a variety of other agents that induce the expression of MT-1 and MT-2 [15, 16].

This laboratory demonstrated that MT-3 expression was not confined to the neural system, with the finding that MT-3 mRNA and protein could be localized to the tubular elements of the human nephron [17, 18]. This laboratory extended the findings in the human kidney to studies of MT-3 expression in mortal and immortal cell culture models of human proximal tubular cells (HPT and HK-2). These studies implicated MT-3 expression in mediating the cell’s choice between apoptotic and necrotic cell death when exposed to Cd^2+^ [19, 20] and in the regulation of the epithelial-mesenchymal transition (EMT) in renal epithelial cells [21–23]. Outside the renal system, MT-3 expression has been limited to an examination of its expression in several human cancers. Elevated expression of MT-3 was demonstrated in bladder, breast, prostate, and non-small cell lung cancer [24–29] and decreased expression was seen in gastric cancer, esophageal adenocarcinoma, and squamous cell cancer [30–32]. A major reason limiting the interest in the study of MT-3 is that the exact function of MT-3 is unknown. The MT-3 protein has no enzymatic activity, and its lack of induction by stress stimuli has not afforded it a potential role in mediating heavy metal toxicity. The ability of MT-3 to participate in Zn^2+^ transfer to other proteins is assumed, but similar to MT-1 and MT-2 is not conclusively shown *in situ*. In the present study, an examination of the binding partners and their localization was undertaken as an initial step in understanding how stable transfection of MT-3 into HK-2 cells could alter vectorial active transport and cell morphology. Vectorial active transport often manifests in monolayer culture of transporting epithelium as dome structures, regional areas of trapped fluid due to transepithelial fluid transport, a property of the HK-2 proximal tubule cell line that is dependent upon the expression of MT-3 [21].

## Materials and methods

### Cell culture

HK-2 cells were purchased from ATCC and the STR authentication was provided by the company. Stock cultures of HK-2 and HK-2 cells transfected with the MT-3 gene, (HK-2(MT-3)) were maintained under serum-free conditions as previously described [17, 21, 33]. Briefly, cells were fed fresh growth medium every 2–3 days consisting of a 1:1 mixture of Dulbecco’s modified Eagles’ medium (DMEM) and Ham’s F-12 growth medium supplemented with selenium (5 ng/mL), insulin (5 μg/mL), transferrin (5 μg/ml), hydrocortisone (36 ng/mL), triiodothyronine (4 pg/mL) and epidermal growth factor (10 ng/mL). HK-2 and HK-2(MT-3) cultures were sub-cultured 1:4 or 1:3 upon reaching 80% confluency or doming (normally 3–7 days post subculture) using trypsin-EDTA (0.05% - 0.02%).

### Transient transfection

HK-2 cells were transiently transfected with 2 μg supercoiled V5-tagged MT-3 DNA construct as described previously [22, 23] using electroporation (Lonza). Sub-confluent cell cultures in the log phase of growth were trypsinized from T-75 flasks, pelleted, and re-suspended in 100 μL of room temperature Nucleofector Solution V (Lonza). Diluted DNA was added and mixed gently before transferring the reaction to the supplied cuvette. Electroporation program V was run, and the entire contents of the cuvette were transferred to 1-well of a 6-well plate containing 2 mL of the media. Cells were allowed 6 hours to recover and attach to the tissue culture-treated matrix before the addition of fresh media supplemented with 30 μM Zn^2+^. Cells were harvested 24 hours following transfection for subsequent analysis.

### Protein isolation

For analysis of protein-protein interactions, HK-2 or HK-2(MT-3-V5) cells were lysed in a gentle lysis buffer in an attempt to limit the dissociation of protein complexes. Cell monolayers were rinsed on ice with cold PBS twice prior to the addition of 100 μL of cold IP lysis buffer (25 mM Tris-HCl, 150 mM NaCl, 1% NP-40, 5% glycerol, pH 7.4) containing EDTA-free protease inhibitors (Sigma) directly to the monolayer. Plates were incubated on ice for 30 minutes with orbital shaking; lysates were collected and transferred to cold microfuge tubes, centrifuged at 10,000*g* for 10 minutes to pellet debris. The protein was quantified by the BCA assay.

### Immunoprecipitation

For co-immunoprecipitation of immune complexes, 1000 μg of HK-2 or HK-2 (MT-3-V5) lysate was first pre-cleared by incubation with 50 μL of Protein A/G magnetic beads (Thermo-Scientific) and rabbit IgG (to eliminate most of the non-specific protein interactions) for 30 minutes at 4°C with gentle end-over-end mixing (10 RPM). The beads were pelleted with a magnet, and the lysates were transferred to a fresh, cold microfuge tube. Next, 20 μg of the anti-V5 antibody (rabbit monoclonal, Abcam #Ab9116) was added to the tube, and the total volume was adjusted to 500 μL. The samples were incubated overnight at 4°C with gentle end-over-end mixing (10 RPM) to allow immune complex formation. The following day, 50 μL of Protein A/G magnetic beads were prepared by first adding 175 μL of IP lysis buffer (described above), mixing, and removal. An additional 1 mL of IP lysis buffer was added, mixed, and removed. Immediately following the final bead wash, the immune complexes were transferred to the tubes containing the prepared beads and incubated at room temperature with gentle end-over-end mixing for 75 minutes to allow the beads to capture the immune complexes. The beads were collected, and the supernatant was removed. The beads were washed 3 times with 500 μL of 25 mM Tris-HCl, 150 mM NaCl, 0.05% Tween-20 (pH 7.4), followed by a final wash with 18Ω H_2_O. Bound proteins were eluted from the antibody-bound beads through incubation with Laemmli Buffer without any reducing agent, with mixing at room temperature for 10 minutes. Controls included were: beads + V5 antibody+ IP lysis buffer (no lysate control); HK-2 (MT-3-V5 lysate) + beads (no antibody control); HK-2 lysate + V5 antibody + beads (no bait control), all controls were processed in an analogous manner simultaneously.

### Western blotting

For Western blotting, protein lysates were prepared in equivalent volumes to contain 20 μg of total protein and mixed with Laemmli Buffer (Bio-Rad) containing either TCEP or β-mercaptoethanol and boiled for 5 minutes at 95°C to reduce and linearize protein. After cooling, samples were loaded onto 4–20% gradient gels (Bio-Rad) and separated by SDS-PAGE. Proteins were transferred to 0.2 μm PVDF membranes using the Trans-blot Turbo transfer apparatus (Bio-Rad). Following the transfer, the membranes were blocked with 5% non-fat milk or bovine serum albumin (BSA) dissolved in 10 mM Tris-buffered saline, 0.1% Tween-20 (TBS-T) for 90 minutes at room temperature. The membranes were incubated with primary antibodies against (human) proteins of interest at dilutions indicated in S1 Table overnight (16 h) at 4°C with orbital shaking. Membranes were washed with TBS-T buffer and incubated with horseradish peroxidase-conjugated secondary antibodies against the species; the primary antibodies were raised for 1 h at room temperature with orbital shaking. Anti-rabbit and anti-mouse antibodies were diluted at 1:3000 (Cell Signaling), and the anti-goat antibody was diluted 1:1000. Proteins of interest were visualized by chemiluminescent HRP detection (Bio-Rad). Primary and secondary antibodies were diluted in the same buffer used for blocking.

### Mass spectrometry

In-gel digests (100 μL) were concentrated by rotary evaporation to 10 μL, desalted, and further purified and concentrated on a Millipore C18 ZipTip (regular) according to the manufacturer’s instructions to a final volume of 2 μL in 50% acetonitrile/0.1% trifluoroacetic acid. A 0.5 μL aliquot was spotted onto a MALDI target plate with an equal volume of á-cyano-4-hydroxycinnamic acid in 50% acetonitrile/0.1% trifluoroacetic acid and analyzed with an AB/Sciex 4800 MALDI TOF/TOF. The MS acquisition was in reflector positive ion mode with a mass range of 800–4000 m/z. Twenty subspectra, at 50 shots per subspectrum, were acquired for a total of 1000 shots per spectrum. The top twenty most intense precursors were selected for MS/MS, starting with the weakest precursor. The MS/MS acquisition was in MS/MS 1kV positive mode with collision-induced dissociation turned off. The MS/MS processing method was with default parameters. Precursor mass window resolution was at 100. Twenty subspectra, at 100 shots per subspectrum, were acquired for a total of 2000 shots per spectrum.

### Protein identification

Peak lists were generated from raw data with AB/SCIEX 4000 Series Explorer version 3.5.28193 using the Peaks to Mascot tool. Default settings were selected except the minimum signal-to-noise was set to 4, and the minimum peak area was set to 200. The MSMS spectra were searched against the human IPI database version 3.52 (72,928 sequences) using Mascot version 2.1 (Matrix Science). Search parameters were set for trypsin as the enzyme allowing one missed cleavage, a peptide error of 1.2 Da, and a fragment ion error of 0.6 Da. Carbamidomethyl (C) was selected as a fixed modification. Variable modifications were N-acetyl (protein), oxidation (M), and pyroGlu (N-term Q). Peptides with Expect values less than 0.05 (ions score of > 38) were accepted outright. Those with Expect values greater than 0.05 were manually inspected before inclusion in the peptide identification list. The relative molecular weights of the identified proteins were consistent with their theoretical masses.

### Immunolocalization

For immunolocalization experiments, the cells were grown in 24 well plates containing 12 mm glass coverslips at 37° C, 5% CO_2_. Cells at confluent density were fixed and stained using previously described procedures [34, 35]. Briefly, cells were fixed in 3.7% buffered, methanol-free formaldehyde (Polysciences, Inc.) for 20 min at room temperature. Coverslips were quenched of free aldehyde with 0.1 M NH_4_Cl for 15 min, followed by permeabilization with 0.1% Triton X-100 for 15–18 min at room temperature. Cells were stained with the primary antibodies for 45 min at 37° C followed by incubation with 2.0 mg/ml of the appropriate Alexa-Fluor-conjugated secondary antibody (Invitrogen) for 45 min at 37° C. Primary antibodies used are listed in S1 Table and all secondary antibodies were Alexa-Fluor conjugates from Invitrogen used at a concentration of 2 μg/mL. They include Alexa Fluor 488 conjugated Goat anti-Rabbit IgG and Donkey anti-Rabbit IgG; as well as Alexa Fluor 568 conjugated Donkey anti-Goat IgG, Goat anti-Mouse IgG, and Donkey anti-Mouse IgG. Dual labeling was performed by incubating with the first primary antibody then its appropriate secondary antibody followed by the same sequence with the next primary antibody and its appropriate secondary antibody. All co-localization experiments were performed in both directions, with antibody1 being used first followed by antibody2 and vice versa on another set of coverslips. All co-localization were found to have positive results regardless of which primary antibody was stained for first. Labeling for F-Actin was performed using a 66 nM concentration of Alexa-Fluor568 tagged phalloidin either alone or after staining for MT-3. Coverslips were mounted in ProLong Diamond anti-fade reagent with DAPI (Invitrogen) for nuclear counter staining. Controls consisted of coverslips treated with PBS instead of primary antibody and was performed for both double and single labeled combinations of secondary antibodies. All controls stained appropriately and had virtually no specific staining when photographed under the same settings that were used for experimental cells. Stained cells were observed using a Leica TCS SPE, DM5500 laser scanning confocal microscope with LAS-X software (Leica Microsystems). Images were obtained by capturing z-slices at the indicated optimal depth per objective lens and processed using LAS-X analysis system software and Adobe Photoshop CS6.

### Molecular docking and visualization

The protein structures for MT-3/MT-1E and the receptors, aldolase A, enolase 1, β-actin, and tropomyosin 3 were prepared *in silico*. The experimentally solved structures of the receptors were taken having PDB IDs: 4ALD (aldolase A), 2PSN (enolase 1), 6NBW (β-actin), and 7KO5 (tropomyosin 3). For human MT-3, only the α-domain was available with an NMR structure with PDB ID 2F5H; and the β-domain was modeled from the mouse. Mouse MT-1 (PDB ID 1DFT) having 70% sequence identity was used, and homology modeling was performed with Modeler [36]. Two domains were connected using a trans configuration with backbone dihedral angle ω≈180° for the peptide bond. The missing residues in the receptors were modeled using Modeler [37]. For comparative studies [23] of the binding partners, MT-1E structure from the AlphaFold [38] database was taken. Since the modelled structures did not have any ligands, the metals were manually added on MT-3 and MT-1E using the technique of comparative modelling via structure superimposition. The modelled structures were energy minimized to achieve a local minima, and to test the accuracy and stereochemical quality of the structures, Procheck [39] and PDBsum [40] were used for validation before and after molecular docking. For docking purposes, the protein structures were prepared in their monomeric form with Pymol [41] and AutoDock4.2 [42] as shown in S1 Fig. Further, atoms included as water, solvents, and alternate occupancies were deleted, and the hydrogen atoms were added. Hawkdock [43] was used for molecular docking. The blind docking method [44] was used for all the receptors, and clusters of the poses with minimum energies were chosen for further analyses. Subsequently, docked complexes of MT-3 with their binding partners were obtained and then visualized for the interactions within the protein-protein interface sites.

### Estimation of binding affinities

The binding affinities between MT-3 and its binding partners were computed using an all-atom energy-based algorithm called Molecular Mechanics/Generalized Born Surface Area (MM/GBSA) [45, 46]. Furthermore, the strength of binding was also evaluated by predicting theoretical dissociation constant K_d_ and binding affinity ΔG by the interfacial residues of protein-protein interactions by using prodigy [47]. The same methods were employed for calculating binding affinities between the mentioned binding partners and MT-1E for a comparative evaluation.

## Results

### Identification of protein interactions with MT-3

**Fig 1:**
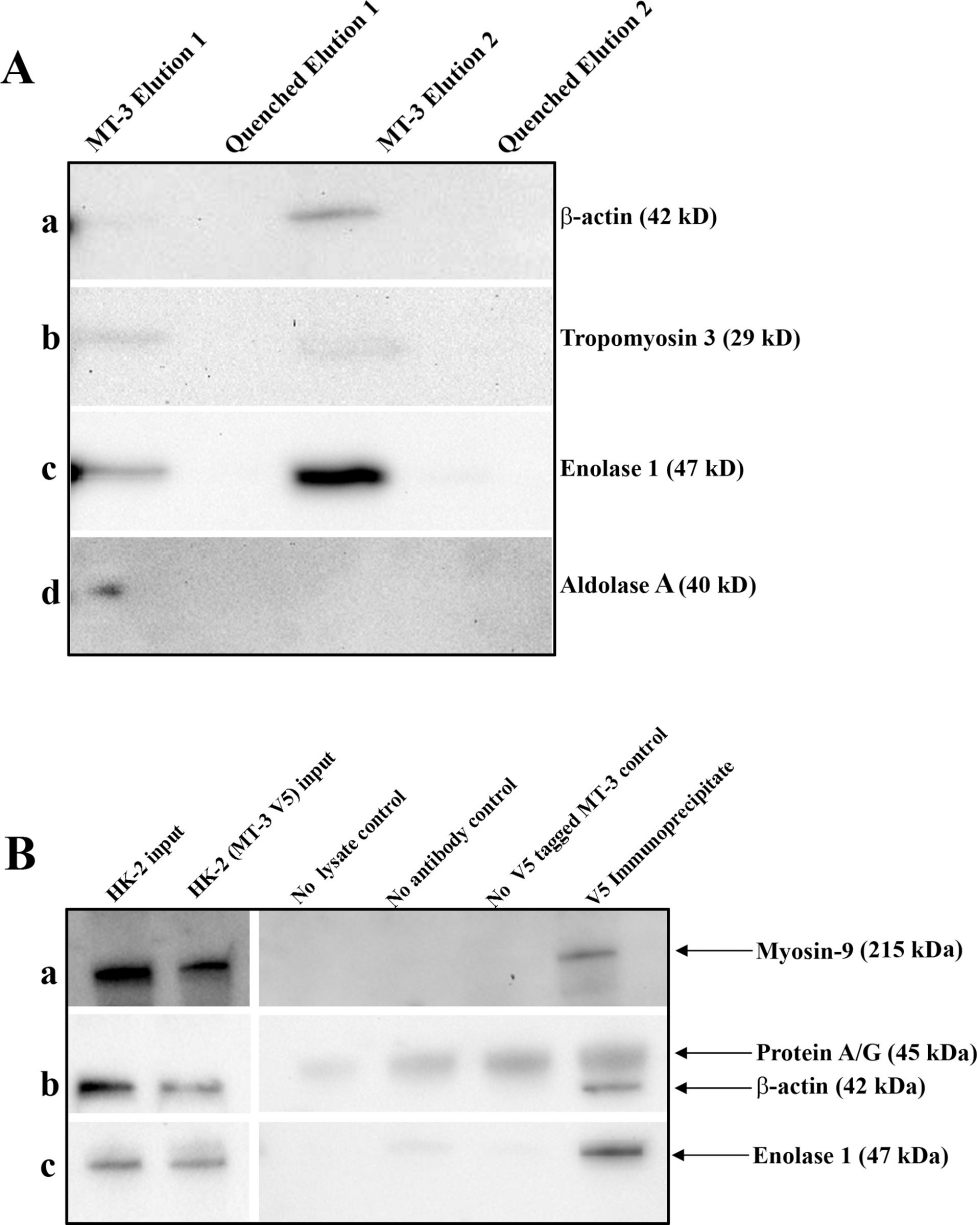
Validation of MT-3 protein interactions by MT-3 mediated pulldown and V5-mediated co-immunoprecipitation. (A). Zn_7_-MT-3 cross-linked agarose beads pulldown proteins isolated from cultured human proximal tubule cells (HK-2) include β-actin (a), tropomyosin 3 (b), enolase 1 (c), and aldolase A (d). (B). MT-3 interacts with myosin-9 (a), β-actin* (b) and enolase 1 (c) in V5-tagged MT-3 expressing proximal tubule cells. To verify the binding of MT-3 to myosin-9, β-actin, and enolase 1 *in vitro*, the V5 antibody was cross-linked to agarose beads through aldehyde linkage and incubated with HK-2 MT-3-V5 cell lysates. Bound proteins were eluted and detected by western blotting. The corresponding protein was not detected in non-transfected control (no V5 tagged MT-3), no antibody control, and no lysate control. *Note: Heat causes Protein A/G to come off the magnetic beads, and the reducing agent dissociates the heavy and light chains of the antibody, resulting in detection via secondary antibody reactivity towards protein A/G.

In order to identify potential protein interactions of MT-3 within the human proximal tubule, 100 μg of purified Zn_7_MT-3 was covalently cross-linked to amine-reactive agarose beads and incubated with lysates (1 mg) isolated from HK-2 cells. The eluates were subjected to SDS-PAGE followed by silver-staining (S2A Fig). Visible protein bands were excised, in-gel trypsinized, and subjected to MALDI-TOF/TOF mass spectrometry (MS/MS), revealing β-actin, myosin-9, and tropomyosin 3 as putative MT-3 protein interactants (Table 1). Previous studies evaluating MT-3 protein interactions in cultured astrocytes identified enolase 1 and aldolase A as MT-3 interacting proteins [33], leading us to evaluate these proteins in addition to those identified by MS/MS. Following the confirmation that proteins were present in eluted fractions, eluates were subjected to SDS-PAGE followed by western blotting (Fig 1Aa–1Ad) to probe for the individual protein interactants.

**Table 1:** Identification of MT-3-binding proteins by MT-3 affinity chromatography.

Symbol	Name	Mass (kD)	Unique peptides	Sequence coverage
MYH9	Myosin heavy chain-9, non-muscle	228	16	12%
ACTB	Actin, cytoplasmic 1	42	7	26%
TPM3	Tropomyosin alpha-3 chain	32	3 (5)*	14%
TPM1	Tropomyosin alpha-1 chain	29	1 (3)*	7%

Purified human Zn_7_-MT-3 was cross-linked to agarose beads and incubated with protein extracts from HK-2 cells. After washing in incubation buffer, the bound proteins were eluted in a high salt elution buffer, dialyzed, and subjected to SDS-PAGE followed by silver staining. Selected stained bands were excised and subjected to in-gel trypsin digestion followed by MS/MS. The protein identities are indicated. All MS/MS were manually validated.

*Numbers in parentheses indicate unique peptides plus peptides shared by TPM3 and TPM1.

### V5-mediated immunoprecipitations in MT-3 expressing HK-2 lysates

To further support the protein-protein interactions observed through pulldowns, we generated mutant cell lines either stably or transiently expressing MT-3. The MT-3 antibody is generated against the hexapeptide C-terminal insert not present in any other metallothionein isoforms. We have hypothesized the C-terminal domain of MT-3 to be the region of protein-protein interactions, as this feature distinguishes MT-3 from MT-1,-2, and -4. This becomes problematic for co-immunoprecipitation studies if the C-terminal domain is blocked by the interactions that are being determined. For these reasons, the stop codon of MT-3 was removed to allow translation of a V5 tag to aid in immunoprecipitation, Western blotting, and immunofluorescence. V5 is a relatively small tag in comparison to most other commercially available protein tags, adding an additional 4 kD. Prior to performing immunoprecipitation, lysates were tested for the presence of MT-3-V5 by Western blotting (S2B Fig). We also confirmed the lack of V5 signal in MT-3 null lysates. Interestingly, cells supplemented with 30 μM Zn^2+^ in the growth medium six hours post-transient transfection contained more MT-3 protein than those supplemented with the normal growth medium (S2B Fig). MT-3 is not inducible by Zn^2+^, so this observation probably correlates to the increased stability of fully Zn^2+^-bound MT-3 compared to apo-MT-3 or other less metallated forms of MT-3, resulting in less turnover of this protein. Co-immunoprecipitation of V5-MT-3 complexes and subsequent western blotting confirmed myosin-9, β-actin, and enolase 1 (Fig 1Ba–1Bc) as MT-3 interacting proteins in the proximal tubule.

### Intracellular localization of MT-3 in HK-2(MT-3) cells

**Fig 2:**
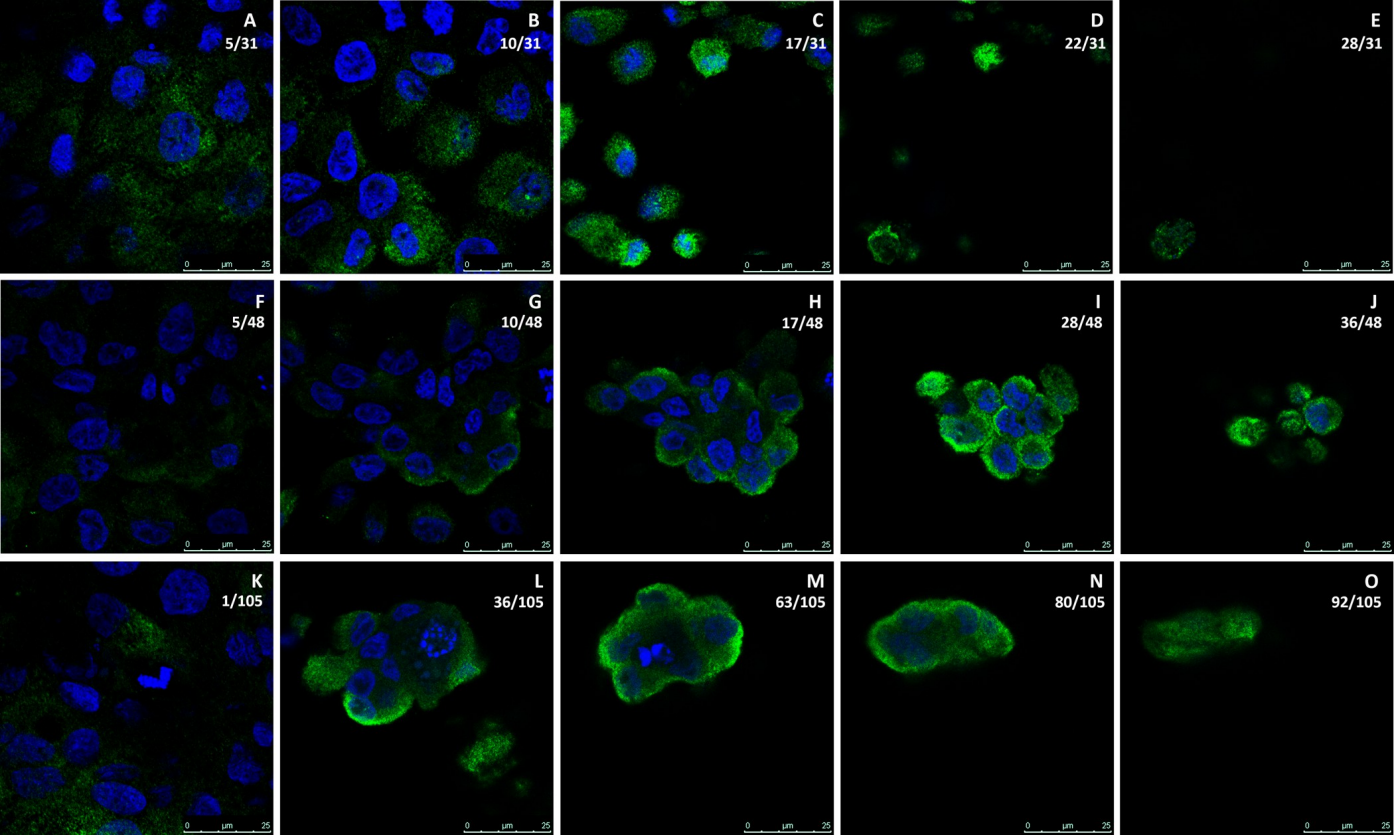
Intracellular localization of MT-3 in a doming population of HK-2(MT-3) cells. MT-3 (green) is seen in a non-doming monolayer (A-E), a moderately tall dome (F-J), and in a taller dome structure (K-O). Nuclei are stained with DAPI (blue). The z-section level (0.35 μm depth) relative to the total number of z-sections through that region is indicated as a ratio in the upper right corner of each image. The scale bar at the bottom right of each image shows increments of 5 μm up to 25 μm.

**Fig 3:**
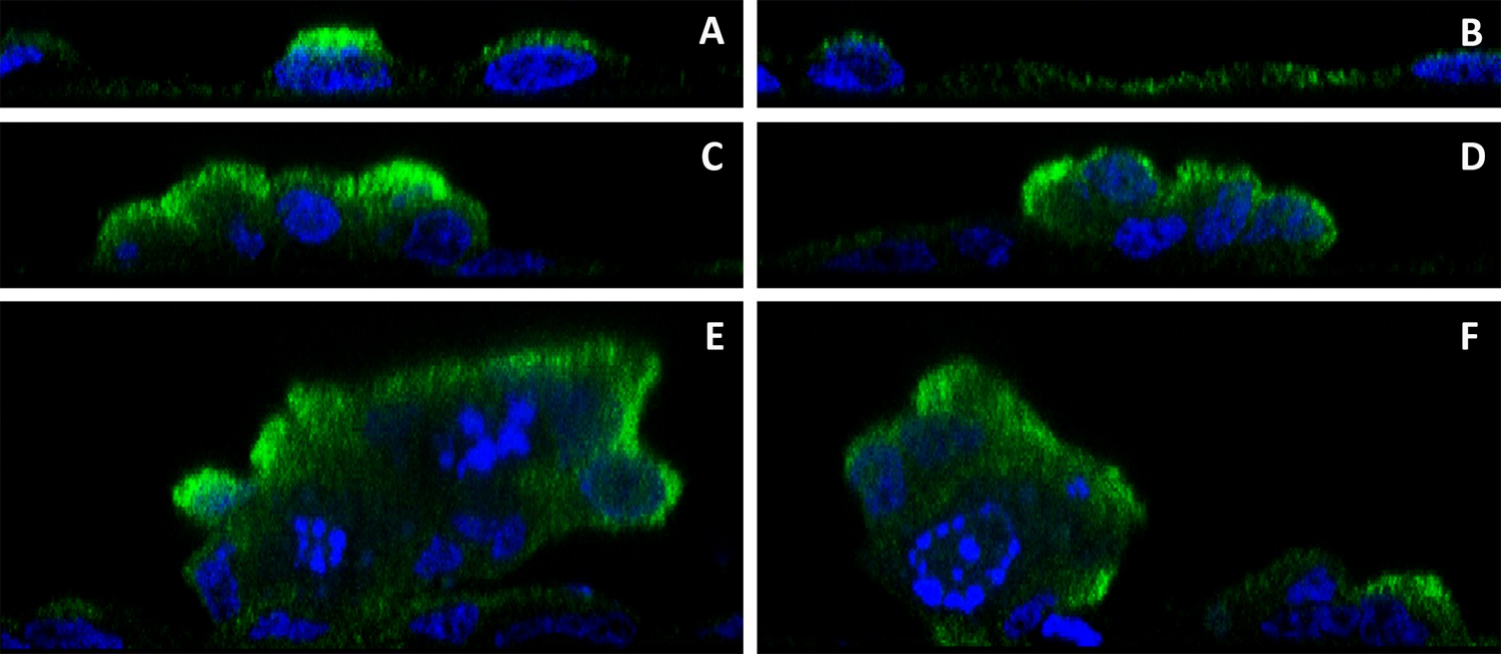
Orthogonal images of MT-3 localization. MT-3 (green) and nuclei (blue) are seen in orthogonal sections along the x-axis (A, C and E) and y-axis (B, D and F) for same 3 fields shown in Fig 1. The non-doming monolayer (A and B), the moderately tall dome (C and D) and the taller dome structure (E and F) are all shown.

Previous studies from this laboratory on human proximal tubule cells have demonstrated that expression of MT-3 is critical for these cells to have vectorial transport and hence the capacity to form domes *in vitro* [18, 21–23]. In this study, the intracellular localization of MT-3 was determined in HK-2 cells that were previously transfected with the MT-3 gene [21]. The localization of MT-3 was determined in both the non-doming cell monolayer as well as within dome structures using laser scanning confocal microscopy. Results showed that in the non-doming monolayer the vast majority of MT-3 localizes diffusely within the cytoplasm (Fig 2A–2E). In many cells, the diffuse cytoplasmic localization of MT-3 also correlated with the apical portion of the cell above the nucleus and was rarely, if ever, found with heavy fluorescent staining at the basal portion of the cell beneath the nucleus (Fig 3A and 3B). Conversely, in the doming portions of the monolayer, the vast majority of MT-3 is localized to the periphery of the cells and, in particular, for larger dome structures to the periphery of the dome itself (Fig 2F–2O). In intermediate domes, as shown in Figs 2F–2J, 3C and 3D, MT-3 was often found localized to the periphery of the dome as well as to the apical aspects of the individual cells. However, within taller domes, MT-3 was often found with high fluorescence intensity, concentrated at the lateral aspects of the dome cell periphery (Figs 2L–2N, 3E and 3F) and also diffusely at the apical aspect of the dome (Fig 2O). Results also showed that nearly all dome structures appear to have low levels of cytoplasmic or basally localized MT-3, with the majority of MT-3 localizing to the lateral and/or apical aspects of the cells within domes, which was particularly evident in the orthogonal views of the dome structures (Fig 3C–3F). These findings are further supported by viewing the fields using three-dimensional analysis of scanning images (S3 Fig) which show that MT-3 is present on the periphery of the dome, and very little is visualized within the dome structure itself.

### Intracellular localization of F-actin in HK-2(MT-3) cells

**Fig 4:**
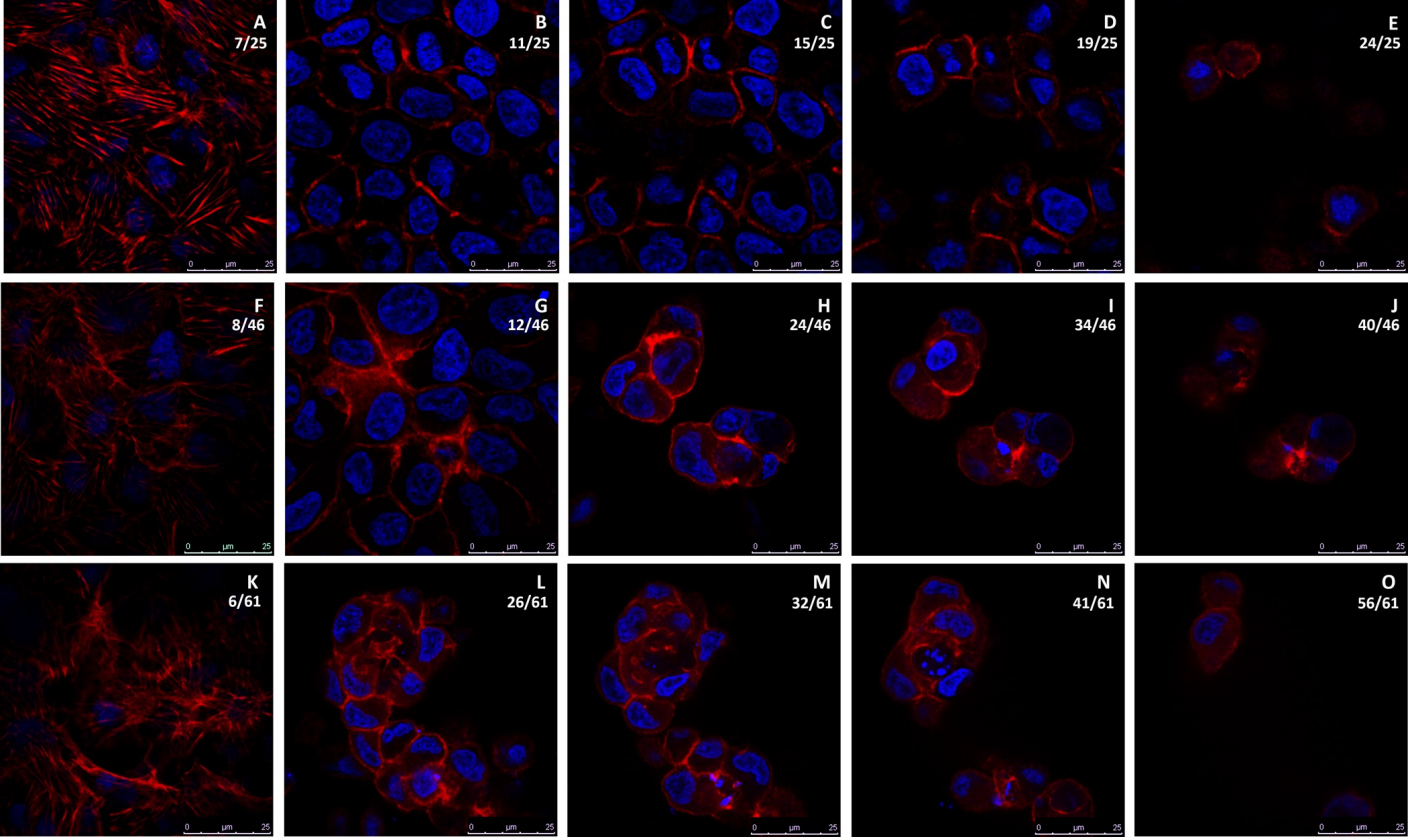
Intracellular localization of F-actin in a doming population of HK-2(MT-3) cells. F-actin (red) is seen in a non-doming monolayer (A-E), a moderately tall dome (F-J), and in a taller dome structure (K-O). Nuclei are stained with DAPI (blue). The z-section level (0.35 μm depth) relative to the total number of z-sections through that region are indicated as a ratio in the upper right corner of each image. The scale bar at the bottom right of each image shows increments of 5 μm up to 25 μm.

**Fig 5:**
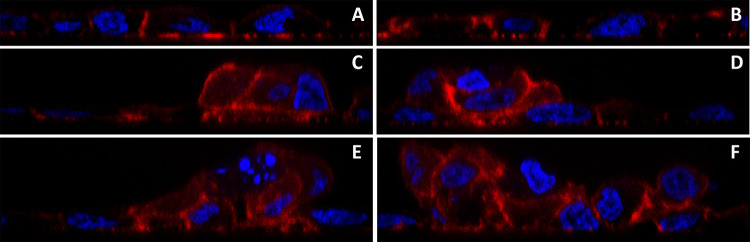
Orthogonal images of F-actin localization. F-actin (red) and nuclei (blue) are seen in orthogonal sections along the x-axis (A, C and E) and y-axis (B, D and F) for same 3 fields shown in Fig 4. The non-doming monolayer (A and B), the moderately tall dome (C and D) and the taller dome structure (E and F) are all shown.

Considering the peripheral localization of MT-3 in dome structures, the localization of filamentous actin (F-actin), which is the key cytoskeletal element in driving cellular shape, was investigated. Results showed that HK-2 (MT-3) cultures had very high fluorescent staining for F-actin stress fibers localized through the basal aspects of the cell along with heavy staining in the lateral localization and yet very little apical concentration (Figs 4 and 5). F-actin was found to rim the cellular periphery regardless of whether or not the cells were associated with domes (Fig 4B–4D, 4G–4I, and 4L–4N). The heavy staining of F-actin not only on the dome periphery but also cellular periphery within the dome (Figs 4G–4J, 4L–4O and 5C–5F) is in stark contrast with MT-3, which was found primarily associated with only the dome periphery. Using the same 3-D analysis as for MT-3 localization, F-actin was confirmed to be exhibit heavy staining at the basal and lateral aspects of the plasma membrane (S4A, S4E and S4I Fig), and little to no F-actin was present on the apical surface so that the top-down view of the domes gave little dimensional orientation (S3B, S3F and S3J Fig). Likewise, 3-D wedges cut from the domes showed very high fluorescent staining and localization of F-actin inside the dome itself (S4C, S4D, S4G, S4H, S4K and S4L Fig). Although there was a strong presence of F-actin within the dome, the heavy staining of F-actin on the lateral edges of the dome periphery was similar to the localization of MT-3. This similarity along with the previous results showing that MT-3 is imperative to dome-formation in these cells is indicative that MT-3 may associate with the actin cytoskeleton either directly or indirectly in order for the HK-2 cells expressing MT-3 to have polarized, vectorial-active transport.

### Co-localization of MT-3 with identified binding partners

**Fig 6:**
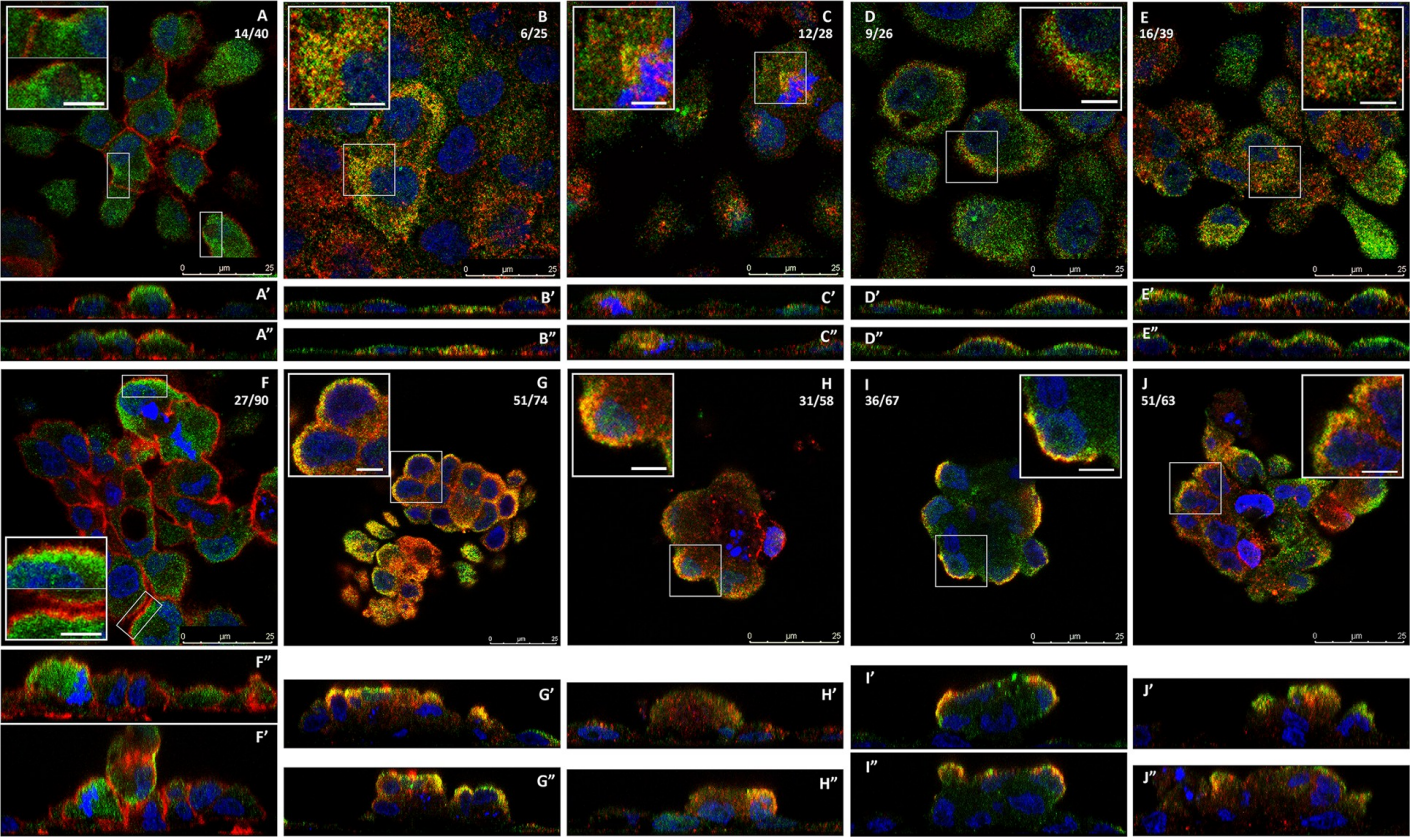
Intracellular co-localization of MT-3 and its binding partners. Images of MT-3 co-localization with the identified binding partners are shown in individual z-sections (0.35 μm depth) for the non-doming monolayer (A-E) and a doming region (F-J). Larger panels have the 25 μm scale bar indicated on the bottom left, and there is an inset indicated with a higher magnification image that contains a 5 μm scale bar. Respective orthogonal views are shown for each image and are indicated (’) for scanning along the x-axis and (") for scanning along the Y-axis. In each panel MT-3 staining is shown in green and the binding partners in red: F-actin (A and F); myosin-9 (B and G); tropomyosin 3 (C and H); enolase 1 (D and I); aldolase A (E and J). The z-section level is shown as a ratio relative to the total z-images taken for that panel.

Based on the immunoprecipitation and mass spectrometry identification of MT-3 binding partners, laser scanning confocal microscopy was used to determine intracellular co-localization. MT-3 was found to co-localize to F-actin at the cell periphery in the non-doming monolayer as well as within dome structures (Fig 6A and 6F, respectively). Overall, the co-localization was minimal in the x/y planes with MT-3 generally being internal to the more peripheral F-actin, but co-localization was observed in multiple fields and in both Z-sections and in the orthogonal views (x/z and y/z planes).

Two classical F-actin binding proteins, myosin-9 and tropomyosin 3, were identified to be MT-3 binding partners, and the level of MT-3 co-localization with these proteins was also assessed. Both myosin-9 and tropomyosin 3 were found to co-localize with MT-3 in the non-doming monolayer (Fig 6B and 6C, respectively) as well as in dome structures (Fig 6G and 6H, respectively). However, the level of co-localization of these two actin-binding proteins with MT-3 was noticeably different. Myosin-9 was found to be co-localized with MT-3 in the perinuclear cytoplasm, often apically, in most cells of non-doming monolayers (Fig 6B, 6B’ and 6B"). In the dome structures, MT-3 and myosin-9 were found to be have a high degree of co-localized at the lateral dome periphery in nearly all cells associated with domes (Fig 6G, 6G’ and 6G"). Tropomyosin 3, was found to co-localize with MT-3 in the cytoplasm of some cells of both the non-doming monolayer and at the dome-periphery (Fig 6C and 6H, respectively). In addition to actin-binding proteins, MT-3 was found to bind to two glycolytic proteins, enolase 1 and aldolase A. Confocal microscopy experiments showed that both enolase 1 and aldolase A had some co-localization with MT-3 in the perinuclear region of the cytoplasm, particularly along the apical aspects of the cells, in the non-doming monolayer (Fig 6D and 6E respectively). However, in domes, MT-3 and enolase 1 showed a high degree of and nearly complete co-localization at the upper lateral periphery of the dome (Fig 6I, 6I’ and 6I"). Aldolase A was also found to co-localize with MT-3 to a high degree along the dome periphery, although not as completely as enolase 1 (Fig 6J, 6J’ and 6J").

**Fig 7:**
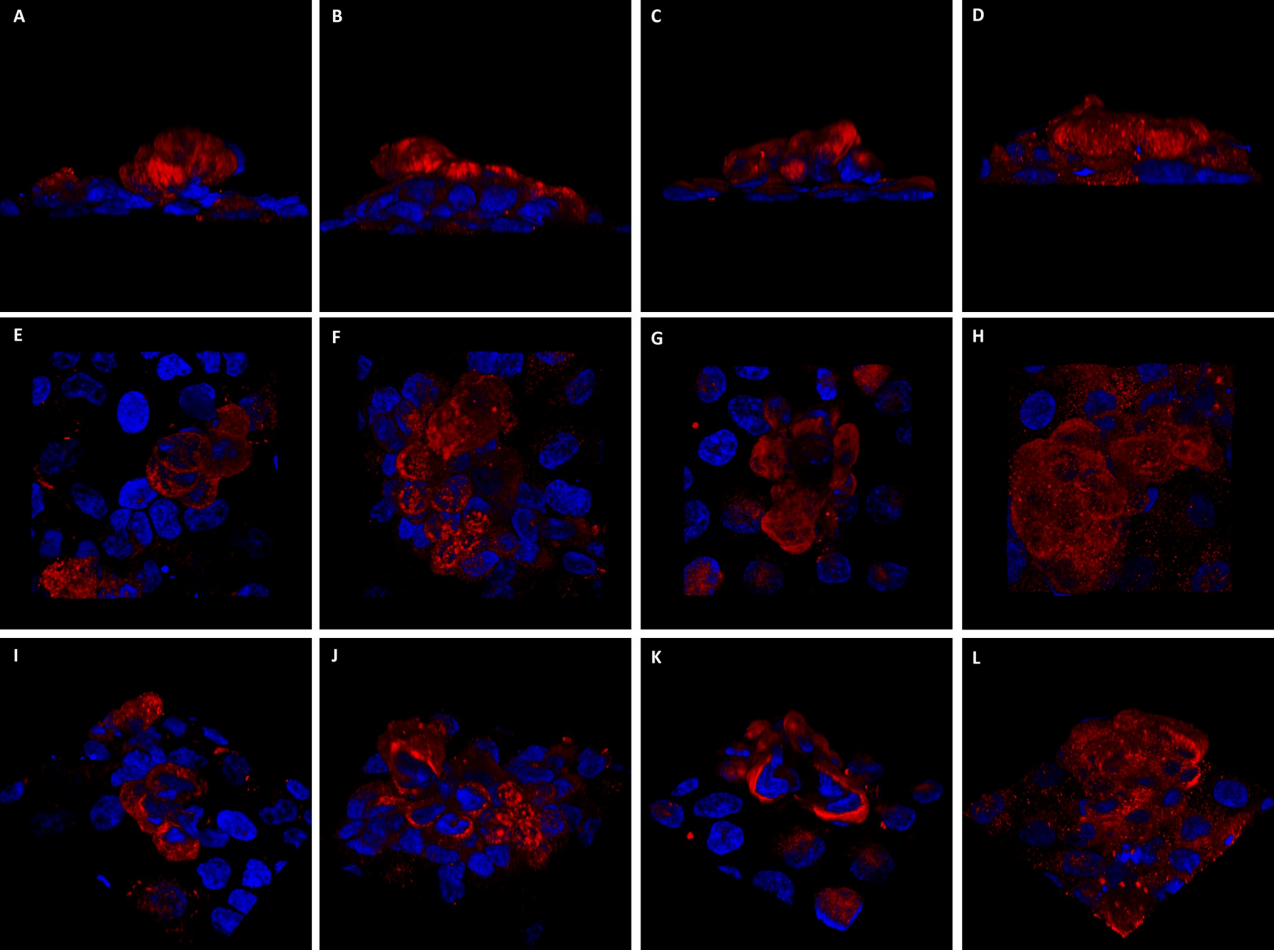
Three dimensional projection of the intracellular localization for the MT-3 binding partners. Each of the binding partners are shown in red: Myosin-9 (A, E and I); tropomyosin 3 (B, F and J); enolase 1 (C, G and K); and aldolase A (D, H and L) and nuclei are shown in blue. The 3 different 3D views shown are side angle view (A-D), apical surface from the top down (E-H), and a portion of the field cut away in a 3D slice (cut in the X, Y, and Z planes) are shown in (I-K). All panels shown are for a representative dome structure.

The localization of each the MT-3 binding partners within the 3-D monolayer was determined to better comprehend doming structures. Unfortunately, 3-D analysis does not provide the resolution or processing needed for determining co-localization of binding partners, however, information on the localization of the individual proteins in the 3-D dome does provide an additional level of understanding. The binding partners, myosin-9, tropomyosin 3, enolase 1 and aldolase A, were found to have a high degree of lateral staining of the dome periphery (Fig 7A–7D, respectively) as well as substantial apical staining in the top-down viewpoint (Fig 7E–7H). However, when a 3-D slice of the dome is cut away from the image offering a view inside the dome, more differences in the localization of these proteins emerge. Myosin-9 and tropomyosin 3 have high degree of peripheral staining but also contain some lighter staining along the plasma membrane and within the dome cells (Fig 7I and 7J). Enolase 1 had very high peripheral staining along the outer face of the dome with very little inside the dome (Fig 7K). This staining pattern is nearly identical to what was observed for MT-3 in the 3-D dome projections (S3 Fig). Conversely, while some aldolase A is concentrated along the dome periphery, there is substantial staining found in punctate structures through the cytoplasm as well as through the depth of the dome (Fig 7L).

Additional views of MT-3 and myosin, enolase 1, and aldolase A can be seen in S5–S7 Figs. These panels provide additional views of the high degree of MT-3 co-localization in the non-doming monolayer and as well as in the dome structures themselves.

### Estimation of protein-protein interactions

The MT-3 binding partners that displayed physical interactions (Fig 1A) with the protein in the above experiments were taken for further analyses. The top ten conformations from molecular docking were evaluated, and clusters of poses with minimum energies (MMGBSA) were taken into account for protein-protein interactions. Table 2 lists two models of each binding partner; their docking scores, binding affinities, K_d_, and ΔG values were calculated for estimation of their binding strengths. The surface representation of these MT-3 poses with the individual proteins are shown in S8 Fig. Moreover, various types of inter-residue interactions between the proteins were explored; these are given for all four complexes in S9 Fig. The insert sequence EAAEAE starting from position 55 to 60 exists in the α-domain of MT-3. Here, with these interactions, all the binding proteins are seen to be interacting with the α-domain of MT-3, especially three binding proteins (Enolase, Tropomyosin, β-Actin) were seen to be interacting with the insert sequence of MT-3. In all of the proteins, several of the amino-acids that are only present in MT-3 and not in MT-1/2 also contributed to protein-protein interactions.

**Table 2:** Protein-protein molecular docking scores obtained for the best two conformations of MT-3 and its binding partners.

Protein	Complex Model	Docking Score (Hawkdock)	MMGBSA Binding Affinity (kcal/mol)	K_d_ (M) at 25°C	Interfacial Contacts ΔG (kcal/mol)
ALDOA	1	-3303.8	-19.95	1.4E-07	-9.3
	2	-3534.4	-18.12	4.0E-10	-12.8
ENO1	1	-3755.3	-7.25	2.5E-07	-9.0
	2	-3578.3	-7.67	4.3E-09	-11.4
ACTB	1	-3374.3	-7.54	6.0E-07	-8.5
	2	-3273.4	-7.44	3.4E-08	-10.2
TPM3	1	-2989.4	-25.72	1.4E-06	-8.0
	2	-2612.1	-14.41	2.1E-07	-9.1

The ligand-protein is MT-3, and the binding partner is considered as the receptor-protein. The best two conformations of MT-3 are considered, and the parameters that define their binding strengths are given.

**Fig 8:**
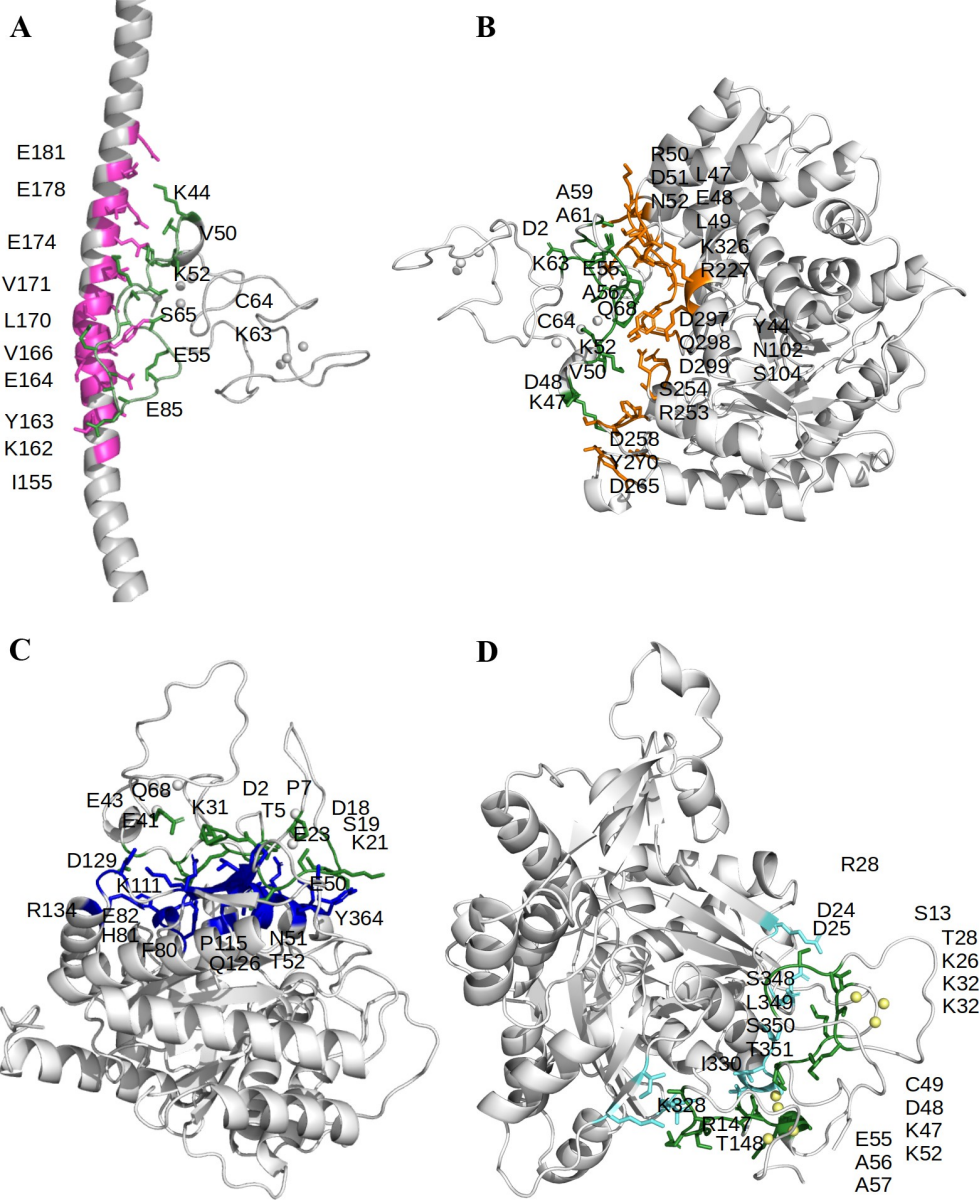
Inter-biomolecular interactions. The surface residues that are taking part in the interactions and making contacts between MT-3 (green) and the proteins (A) tropomyosin 3, B) enolase 1 (C) aldolase A, and (D) β-actin. All residues are colored and labeled.

In the case of tropomyosin 3, there were many docked conformations clustered on two positions, and the binding affinity of the best conformation was -25.72 kcal/mol. Further, the interacting residues of both receptor-protein and ligand-protein are shown in Fig 8A. Importantly, three hydrogen bonds, two salt bridges, and several non-bonded interactions were seen with the α-domain; moreover the insert sequence residues E55, A56, E58, and A59 were involved in these interactions. For enolase 1, the favorable binding affinities were seen for poses that were bound near the catalytic site of the enzyme and Fig 8B shows the best conformation with binding affinity of -7.67 kcal/mol. Here, four hydrogen bonds, one salt bridge, and several non-bonded interactions were seen with α-domain; moreover, E55 and A59 from the insert sequence was also involved. Similarly, in the case of aldolase A, it was seen that most of the top conformations were for poses that were in the vicinity of the active site and the binding affinity of the best conformation was -19.95 kcal/mol as shown in Fig 8C. In this, six hydrogen bonds, two salt bridges were observed with MT-3. Lastly, for β-actin (Fig 8D), the most favorable poses were in the prevalent target-binding cleft [48] and the binding affinity was -7.54 kcal/mol. In this, one hydrogen bond and one salt bridge was observed with several non-bonded contacts with the α-domain involving E55, A56, and A57. Overall, it was observed that all the binding partners showed substantially strong strength of binding.

When comparing the docking simulation results of MT-3 and MT-1E (S10 Fig), it was noted that in Aldolase A, all the parameters of docking score, MMGBSA binding affinity, ΔG, and K_d_ showed higher affinity towards MT-3. Moreover, the proteins Enolase 1, β-actin, and Tropomyosin showed stronger affinity towards MT-3 as indicated by the outcome of parameters of docking score, ΔG, and K_d_ as compared to the MT-1E with exception of the values for the MMGBSA binding affinity. Overall, the discovered binding partners of MT-3 showed stronger binding affinity with MT-3 due to the contribution of α-domain in residue-residue interactions, especially with the residues that are only present in MT-3 (S9 Fig).

## Discussion

The MT-3 isoform of the MT gene family was first identified as a tissue specific family member with expression confined to the neural system [11–13]. Interest was generated in MT-3 since it was shown to be down-regulated in Alzheimer’s disease brains and to possess a neural growth inhibitory activity. It was also shown to be structurally distinct from the two highly studied MT-1 and MT-2 members of the gene family in having a unique 6 amino acid C-terminal sequence and a Thr in the N-terminal region. This laboratory expanded the tissue distribution of MT-3 with the demonstration that MT-3 was expressed in the tubular elements of the human kidney [18]. These studies were extended further by the laboratory to include an analysis of MT-3 expression in mortal and immortal cell culture models (HPT and HK-2) of the human proximal tubular cells [49, 50]. Using these HK-2 and HPT cell culture models, it was demonstrated that MT-3 played a role in cellular differentiation as noted by the ability of the HPT cells, but not HK-2 cells, to express vectorial active transport [21–23]. Vectorial active transport in cultured epithelial cells is noted by the light microscopic demonstration of "doming structures" within the cell monolayer [51, 52]. The "doming structures" are raised, out-of-focus, areas where fluid has become trapped underneath the monolayer owing to active transport of ions and water across the cell monolayer in an apical to basolateral direction. This, in turn, traps a bubble of fluid between the cell layer and the culture dish, forcing local detachment of the monolayer from the plastic surface, forming a raised area with an underneath reservoir of accumulated fluid. The formation of "domes" requires functional plasma membrane polarization, a basolateral Na^+^, K^+^-ATPase, tight junctions between adjacent cells, and vectorial Na^+^-coupled active transport. This laboratory has shown that the stable transfection of the MT-3 gene into the HK-2 cells, results in the HK-2 cells gaining the ability to form "domes" with an overall increase in epithelial morphology [21–23].

The localization of MT-3 in the HK-2(MT-3) cells supported a possible direct structural role for MT-3 in re-establishing vectorial active transport by the parent HK-2 cells. Examination by confocal microscopy showed that MT-3 was concentrated at the periphery of the cells that formed domes, and for larger doming structures, to the periphery of the dome itself. In non-doming areas of the monolayer, MT-3 was localized diffusely in the cytoplasm. In addition, in dome structures, the cells appeared to have lower levels of cytoplasmic MT-3, supporting the possibility that MT-3 migrates and becomes localized to the lateral and/or apical aspects of the cells that form domes. Three of the five binding partners identified to interact with MT-3 are also consistent with a structural role for MT-3 in promoting vectorial active transport, as β-actin, myosin-9, and tropomyosin 3 are all associated with the structural integrity of cells. Co-localization studies of MT-3 with these 5 proteins were also supportive that MT-3 could play a structural role in re-establishing vectorial active transport in the HK-2 cells. A major difference between the parental HK-2 cells and the HK-2 cells stably transfected with MT-3 is the formation of tight junctions in the stably transfected cells. As such, an interaction of MT-3 with components of the cytoskeleton would be consistent with an increase in cell polarity and the formation of the junctional complex between cells. Overall, the finding that MT-3 can associate with β-actin, myosin, and tropomyosin-3 are consistent with a role for MT-3 in altering cell differentiation to facilitate vectorial active transport.

As tropomyosin-3, β-actin, and aldolase A showed direct physical associations with MT-3 in our studies, the structural point of view of these proteins was examined in the cellular organization to comprehend their functional role. It is known that β-actin forms a complex with tropomyosin-3 which is a part of the microfilament and the cell cytoskeleton [53]. Aldolase A also binds with β-actin to participate in the dynamics of the cytoskeleton and contribute to functions such as cell mobility and cell shape [54, 55]. The colocalization results and the molecular docking studies for each of these cytoskeleton proteins with MT-3 suggest that MT-3 aggregates into a biomolecular assembly with tropomyosin-3 and β-actin complex in the apical region and potentially Aldolase A and β-actin complex as well. These inferences involving the MT-3 interactions with the cytoskeleton proteins strengthen the role of MT-3 as a major player in the biological processes of cell motility, cell structure, and cell division.

The above evidence suggests that MT-3 and its binding partners potentially interact to influence renal cell differentiation and vectorial active transport; however, it is challenging to define the mechanism of how this binding might occur. *In silico* studies presented here reflect an idea of the structure-function relationship exhibited by the residues that contribute to the protein-protein interactions. The MT-3 protein stands out from all other human metallothioneins by two major differences discussed here. First is the unique N-terminus sequence TCPCP at the position 5 to 9 present in the β-domain, and second is a C-terminus stretch of loop insert with the sequence GEAAEAEA at the position 54 to 61 in the α-domain. Both of the structural proteins, β-actin and tropomyosin, had strong binding affinity (ΔG) with MT-3. Moreover, aldolase and enolase-1 showed even stronger binding affinities due to the presence of extra number of hydrogen bonds and salt bridges between the interface of the proteins. All of the binding partners interacted with the α-domain of MT-3, as seen in the docked protein complexes. β-actin and aldolase were seen to have several contacts with both β- and α-domain of MT-3 while tropomyosin and enolase only interacted with the α-domain of MT-3.

A previous study had identified Rab3a as an MT-3 interacting protein in the brain [56]. Rab3a is involved in the cycling of zinc through zinc-containing vesicles, being released into the synaptic cleft during neurotransmission followed be reuptake of zinc and reloading into these synaptic vesicles. MT-3 binds to the GDP form of Rab3a and not of the GTP-bound form, and this interaction is thought to regulate trafficking of these vesicles. Rab3a was not found to be expressed in our renal epithelial cells when global gene expression analysis was used nor is expressed in the kidney as indicated from the Human Protein Atlas. Other Rab isoforms are possible and there are approximately 60 isoforms of these proteins, most of which are involved in vesicular trafficking [57]. We did not detect any Rab isoforms in our experiments but cannot rule out involvement of these unique GTPase proteins in the regulation of vectorial active transport and/or the polarity of proximal tubule cells. There does not appear to be much exocytosis from the apical side of proximal tubule cells, but endocytosis of protein occurs extensively in this region of the nephron [58].

Previous studies on MT-3 in the neural system show that both β-actin and myosin-9 are binding partners for MT-3 [33, 59]. Two subsequent studies in astrocytes provide further evidence of the binding of MT-3 with β-actin [60, 61]. Both studies implicate MT-3 in promoting actin polymerization. The first in epidermal growth factor-induced c-Abl activation in astrocytes and the second in the endocytosis of amyloid β from astrocytes. Both investigations suggest that MT-3 can associate with and promote, polymerization of F-actin and that this interaction requires the unique N-terminal sequence of MT-3. Furthermore, the above studies and additional studies of the structure-functional relationships of MT-3 [62–65] advance that the formation of a tightly bound metal-thiolate cluster of MT-3 yields a characteristic conformational change of the TCPCP—terminal sequence that provides a potential interface for protein/protein interactions with MT-3. In agreement with the above, a fragment of MT-3 containing the TCPCP motif was shown to interact physically with F-actin [60]. In the present study, the challenge with an N-terminal TCPCP binding site is that a previous study has shown the C-terminal sequence of MT-3 is required for HK-2 cells to gain vectorial active transport and not the N-terminal sequence [23]. Furthermore, it was shown in the study that inserting the C-terminal sequence of MT-3 into the MT-1E gene with subsequent stable transfection into the HK-2 cell line resulted in the cells gaining increased differentiation and vectorial active transport. The human MT-1E gene does not contain the unique N-terminal sequence of MT-3, and protein binding partners have never been previously identified for the MT-1 and MT-2 family members. The best explanation for the above findings is that the unique C-terminal sequence of MT-3 modifies the β-domain of MT-1E to allow protein interactions with the above-identified MT-3 binding proteins. The formation of complexes with actin-binding proteins is not rare; a vast number of such interactions have been identified for this protein [66, 67]. The finding that the unique C-terminal sequence of MT-3 can promote vectorial active transport when inserted into the MT-1E gene is not a unique finding for renal HK-2 cells. The stable transfection of MT-1E containing the unique C-terminal sequence of MT-3 into MCF-7, a breast cancer cell line, has also been shown to induce dome formation in the stable transformants [68]. Together these findings suggest that the unique C-terminal insert of MT-3 allows an interaction to be formed with β-actin, myosin-9, and tropomyosin 3 and that this promotes vectorial active transport and an increase in the differentiated function of the epithelial cells. Further, it is observed in the *in-silico* docking models of MT-3 with the binding partners Enolase, Tropomyosin, and β-Actin that this unique sequence is contributing to the important residue-residue interactions. Enhanced binding scores for MT-3 were not always greater than that of MT-1E as was the case for three MMGBSA simulations but the overall preponderance of these simulation cases shows enhanced binding to MT-3. Different outcomes in these simulations are not uncommon [69, 70]. Simulation of K_d_ and ΔG weigh intermolecular attractive forces to a greater extent than Docking Score and MMGBSA, which place more emphasis on complementary fit and surface topology [71]. An analysis of SARS-CoV-2 spike protein variants in its interaction with ACE2 receptor is another example where each *in silico* approach gave different binding rankings for each *in silico* approach [72].

The remaining two proteins identified as binding partners for MT-3, enolase 1, and aldolase A, are both glycolytic enzymes. Confocal microscopy showed a very high degree and nearly complete co-localization of both enolase 1 and aldolase A with MT-3 at the upper lateral periphery of the domes as well as the periphery of the domes. This suggests that these proteins could also have a role in promoting and maintaining vectorial active transport. Both of these proteins are involved in glycolysis and suggest that one function would be to provide an energy-rich environment for the cells supporting dome formation and vectorial active transport. The cells involved in dome formation can be viewed as having an enhanced need for ATP since the basolateral Na^+^, K^+^-ATPase is required as one element of the active transport process. While primarily known as a ubiquitous cytosolic metabolic pathway, localization of the glycolytic process under certain conditions has been described: localization to nerve terminals under stress [73] and one report of localization of a GAPDH inhibitor leading to decreased apical membrane transport function in renal epithelial cells [74]. A more direct role of enolase and aldolase is suggested by studies that show these two enzymes have roles well beyond their well-known function in glycolysis. Enolase-1 has been shown to localize to the plasma membrane as a plasminogen receptor as well as function as a DNA binding protein in the process of transcription and has involvement in multiple signal transduction pathways [75]. Aldolase A has already been implicated as an actin-binding protein that regulates polymerization of actin [76]. These studies are more suggestive of roles contributing to the organization of subcellular structures, in partnership with MT-3, in the apical region of the cell. Overall, the binding partners identified to interact with MT-3 are consistent with an alteration of the cytoskeleton of the cell necessary to support dome formation and the increased need for energy that would be required to support this process.

Recent studies on MT-3 show brain MT-3 contains both copper and zinc, (copper residing in the N-terminal β-domain and zinc in the α-domain) and that MT-3 is capable of sequestering copper from amyloid. The metal complement of MT-3 is not known for tissues outside of the neurosystem [77, 78]. There are reports that MT-3 can function in zinc acquisition and loading in the immune cells and mucosal epithelial [79, 80]. The metal content of renal MT-3 is currently unknown. Increased metal exposure was unable to increase MT-3 levels in proximal tubule cells despite a near 100-fold induction of MT-1/2 protein levels in mortal cultures of human proximal tubule cells [81]. Thus, the regulation of MT-3 protein levels appear to be distinct from that of the other isoforms of this protein within the kidney. While the protein binding partners of MT-3 in this study were initially performed with a pure Zn_7_-MT protein, the verification of binding in situ by transfection of the MT-3 coding sequence allows MT-3 to acquire metals from the normal cellular milieu and metal pools. Metallothionein is also known to have very dynamic metal binding, participating in ligand exchange reactions, and in the case of MT-3, the binding dynamics have many unique features compared to other MT isoforms, one being decreased stability of the α-domain. Defining an exact role for MT-3 and Zn^2+^ in the present study is difficult since more than 300 enzymes, and 1,000 transcription factors are known to require Zn^2+^ for activity [82]. However, studies on tight junctions in intestinal epithelium have shown an essential role for Zn^2+^ in maintaining tight junctional integrity [83]. The role of Zn^2+^ appeared to be at the level of the transcriptional regulation of claudin and occludin expression and not a direct interaction of Zn^2+^ with the structural proteins of the tight junctions. Metal transfer to or from localize proteins, stabilization of bound metal or even redox process with sulfhydryl groups is suggestive due to the role of MT and MT-3 in particular in these processes, but there are no metals in the area of putative MT-3 interactions in the identified binding partners, and, thus, currently a mechanistic process is elusive in explaining how MT-3 restores epithelial character and vectoral active transport in these cells. With one report of zinc pools regulating actin dynamics in cell migration in PC-3 cells [84], an essential role of MT-3 in this process cannot be ruled out.

The current study has implicated several proteins as partners with MT3 that appear to form structures in the periphery of renal epithelial cells that gives some insight into the ability of MT3 to confer its ability to re-establish epithelial morphology and vectorial active transport. In silico analysis also gives further insight into some of these protein interactions with MT3, but it also needs to be emphasized that the formation of these structures can also occur through the formation of multi-protein complexes in which MT3 binding may be mediated by other yet-to-be identified proteins. Further research is need to establish and characterize these specific protein interactions.

## Supporting information

S1 Table Primary antibodies used for western blotting and immunolocalization. (DOCX)

S1 Raw images Uncropped blots used to generate Fig 1. PDF file containing TIFF images of all raw uncropped Western blot results. **A.** Blots used to generate Fig 1A. **B and C.** Blots used to generate Fig 1B. X indicates lanes not used.
(PDF)

S1 Fig Molecular structures for docking. a). MT-3 and it’s two domains with β- and α-domain linked at K31-K32, the metal atoms (Zn^2+^) shown as orange spheres are encapsulated with Cys shown as pink sticks, sulfur shown in yellow, and each Zn^2+^ is linked with four other atoms forming a tetrahedral geometry. b). Aldolase A with active site residues highlighted in red and labeled. The primary catalytic residues are K229, E187, and K147 and the secondary site residues are R55 and Y363. c). β-actin in its monomeric form containing a Ca^2+^ atom. d) Enolase 1 with active site residues highlighted in red and labeled. The metal binding residues are S40, D245, E293, D318 and the substrate binding site residues include H158, E167, E210, K343, and K394. e). The oligomeric form of β-actin and tropomyosin 3 complex. The actin molecules are shown in the middle of the filament colored by single monomeric entities and tropomyosin 3 is shown in its homo-dimer form on each side of the filament colored in pink. f). Tropomyosin 3 as a single entity.
(TIF)

S2 Fig Silver-stain and MT-3-V5 western blot of a representative co-immunoprecipitation. Co-immunoprecipitation of V5-tagged MT-3 and putative protein interactants. Eluates were subjected to SDS-PAGE and silver-staining (A) or western blotting (B) for MT-3.
(TIF)

S3 Fig Three-dimensional projections of MT-3 intracellular localization. MT-3 (green) and nuclei (blue) are seen in 4 different 3D views. The fields are the same ones shown in Figs 1 and 2. The side angle view is shown in (A, E, I); a view of the apical surface from the top-down is shown in (B, F, J); two different angle views with a portion of the field cut away in a 3D slice (cut in the X, Y, and Z planes) is shown in (C-D, G-H, K-L). The non-doming monolayer (A-D), the moderately tall dome (E-H), and the taller dome structure (I-L) are all shown.
(TIF)

S4 Fig Three dimensional projection of F-actin intracellular localization. Three dimensional projection of F-actin intracellular localization. F-actin (red) and nuclei (blue) are seen in 4 different 3D views. The fields are the same ones shown in Figs 1 and 2. The side angle view is shown in (A, E, I); a view of the apical surface from the a top-down is shown in (B, F, J); two different angle views with a portion of the field cut away in a 3D slice (cut in the X, Y, and Z planes) is shown in (C-D, G-H, K-L). The non-doming monolayer (A-D), the moderately tall dome (E-H) and the taller dome structure (I-L) are all shown.
(TIF)

S5 Fig MT-3 co-localization with myosin-9. MT-3 (green, A and E) and myosin-9 (red, B and F) localization in HK-2 MT-3 cells. Respective orthogonal views are shown for each image and are indicated (’) for scanning along the x-axis and (") for scanning along the Y-axis. Panels C and G show co-localization (yellow) of MT-3 and myosin-9; panels D and H display the same co-localization with the addition of DAPI (blue) for nuclear visualization. Multiple regions of co-localization were found and are shown (I-L).
(TIF)

S6 Fig MT-3 co-localization with enolase 1. MT-3 (green, A and E) and enolase 1 (red, B and F) localization in HK-2 MT-3 cells. Respective orthogonal views are shown for each image and are indicated (’) for scanning along the x-axis and (") for scanning along the Y-axis. Panels C and G show co-localization (yellow) of MT-3 and enolase 1; panels D and H display the same co-localization with the addition of DAPI (blue) for nuclear visualization. Multiple regions of co-localization were found and are shown (I-L).
(TIF)

S7 Fig MT-3 co-localization with aldolase A. MT-3 (green, A and E) and aldolase A (red, B and F) localization in HK-2 MT-3 cells. Respective orthogonal views are shown for each image and are indicated (’) for scanning along the x-axis and (") for scanning along the Y-axis. Panels C and G show co-localization (yellow) of MT-3 and aldolase A; panels D and H display the same co-localization with the addition of DAPI (blue) for nuclear visualization. Multiple regions of co-localization were found and are shown (I-L).
(TIF)

S8 Fig Molecular docking complexes of MT-3 with its binding partners. The figure shows two best poses of bound MT-3. a). Aldolase has binding affinities of -19.95 kcal/mol for grey conformation and -18.12 kcal/mol for magenta conformation of MT-3. It is seen that the best conformations are for poses that were around the active site. b). Enolase 1 has binding affinities of -7.67 kcal/mol for orange conformation and -7.25 kcal/mol for green conformation of MT-3. It is seen that best binding affinities are for poses that are bound near the catalytic site of the enzyme. c). β-actin has binding affinities of -7.54 kcal/mol for green conformation and -7.44 kcal/mol for pink conformation of MT-3. d). Tropomyosin 3 has binding affinities of -25.72 kcal/mol for purple conformation and -14.41 kcal/mol for blue conformation of MT-3. It is seen that many docked conformations clustered on these two positions.
(TIF)

S9 Fig Residue-residue interactions between MT-3 and its binding partners. The number of interactions are given on the top with smaller circle representing MT-3 and larger being the binding partner. The interaction type is labeled and further it is seen that most interactions are with the α-domain of MT-3. The residues that are only present in MT-3 are underlined and the insert loop residues (EAAEAE) are marked with an asterisk (*). A pair-wise alignment of MT-3 and MT-1E is also shown to highlight these amino acids.
(TIF)

S10 Fig Comparison of MT-3 and MT-1E docking models. The protein-protein complexes of the binding partners of MT-3 were compared with docking models of MT-1E using parameters of docking score, MMGBSA affinity, ΔG, and K_d_. a) Aldolase A showed stronger binding with MT-3 as compared to MT-1E in all the parameters; b) Enolase 1, c) β-actin and d) Tropomyosin showed stronger binding of MT-3 with its binding partners as compared to MT-1E on account of docking score, ΔG, and K_d_.
(TIF)

## References

[1] Ziller A, Fraissinet-Tachet L. Metallothionein diversity and distribution in the tree of life: a multifunctional protein. Metallomics. 2018;10: 1549–1559. doi: 10.1039/c8mt00165k 30229264

[2] Stennard FA, Holloway AF, Hamilton J, West AK. Characterisation of six additional human metallothionein genes. Biochimica et Biophysica Acta (BBA)-Gene Structure and Expression. 1994;1218: 357–365. doi: 10.1016/0167-4781(94)90189-9 8049263

[3] West AK, Stallings R, Hildebrand CE, Chiu R, Karin M, Richards RI. Human metallothionein genes: structure of the functional locus at 16q13. Genomics. 1990;8: 513–518. doi: 10.1016/0888-7543(90)90038-v 2286373

[4] Coyle P, Philcox JC, Carey LC, Rofe AM. Metallothionein: the multipurpose protein. Cellular and Molecular Life Sciences CMLS. 2002;59: 627–647. doi: 10.1007/s00018-002-8454-2 12022471 PMC11337511

[5] Petering DH, Mahim A. Proteomic high affinity Zn2+ trafficking: where does metallothionein fit in? International journal of molecular sciences. 2017;18: 1289. doi: 10.3390/ijms18061289 28629147 PMC5486110

[6] Maret W. Metallothionein redox biology in the cytoprotective and cytotoxic functions of zinc. Experimental gerontology. 2008;43: 363–369. doi: 10.1016/j.exger.2007.11.005 18171607

[7] Namdarghanbari M, Wobig W, Krezoski S, Tabatabai NM, Petering DH. Mammalian metallothionein in toxicology, cancer, and cancer chemotherapy. JBIC Journal of Biological Inorganic Chemistry. 2011;16: 1087–1101. doi: 10.1007/s00775-011-0823-6 21822976

[8] Iszard MB, Liu J, Klaassen CD. Effect of several metallothionein inducers on oxidative stress defense mechanisms in rats. Toxicology. 1995;104: 25–33. doi: 10.1016/0300-483x(95)03118-y 8560499

[9] Haq F, Mahoney M, Koropatnick J. Signaling events for metallothionein induction. Mutation Research/Fundamental and Molecular Mechanisms of Mutagenesis. 2003;533: 211–226. doi: 10.1016/j.mrfmmm.2003.07.014 14643422

[10] Vašák M, Meloni G. Chemistry and biology of mammalian metallothioneins. JBIC Journal of Biological Inorganic Chemistry. 2011;16: 1067–1078. doi: 10.1007/s00775-011-0799-2 21647776

[11] Tsuji S, Kobayashi H, Uchida Y, Ihara Y, Miyatake T. Molecular cloning of human growth inhibitory factor cDNA and its down-regulation in Alzheimer’s disease. The EMBO Journal. 1992;11: 4843–4850. 1464312 10.1002/j.1460-2075.1992.tb05590.x PMC556960

[12] Uchida Y, Takio K, Titani K, Ihara Y, Tomonaga M. The growth inhibitory factor that is deficient in the Alzheimer’s disease brain is a 68 amino acid metallothionein-like protein. Neuron. 1991;7: 337–347. doi: 10.1016/0896-6273(91)90272-2 1873033

[13] Palmiter RD, Findley SD, Whitmore TE, Durnam DM. MT-III, a brain-specific member of the metallothionein gene family. Proceedings of the National Academy of Sciences. 1992;89: 6333–6337. doi: 10.1073/pnas.89.14.6333 1631128 PMC49495

[14] Sewell AK, Jensen LT, Erickson JC, Palmiter RD, Winge DR. Bioactivity of Metallothionein-3 Correlates with Its Novel. beta. Domain Sequence Rather Than Metal Binding Properties. Biochemistry. 1995;34: 4740–4747. doi: 10.1021/bi00014a031 7718580

[15] Aoki C, Nakanishi T, Sogawa N, Ishii K, Ogawa N, Takigawa M, et al. Stimulatory effects of 4-methylcatechol, dopamine and levodopa on the expression of metallothionein-III (GIF) mRNA in immortalized mouse brain glial cells (VR-2g). Brain research. 1998;792: 335–339. doi: 10.1016/s0006-8993(98)00239-x 9593981

[16] Hernandez J, Molinero A, Campbell IL, Hidalgo J. Transgenic expression of interleukin 6 in the central nervous system regulates brain metallothionein-I and-III expression in mice. Molecular brain research. 1997;48: 125–131. doi: 10.1016/s0169-328x(97)00087-9 9379832

[17] Hoey JG, Garrett SH, Sens MA, Todd JH, Sens DA. Expression of MT-3 mRNA in human kidney, proximal tubule cell cultures, and renal cell carcinoma. Toxicology letters. 1997;92: 149–160. doi: 10.1016/s0378-4274(97)00049-0 9295238

[18] Garrett SH, Sens MA, Todd JH, Somji S, Sens DA. Expression of MT-3 protein in the human kidney. Toxicology letters. 1999;105: 207–214. doi: 10.1016/s0378-4274(99)00003-x 10355541

[19] Somji S, Garrett SH, Sens MA, Gurel V, Sens DA. Expression of metallothionein isoform 3 (MT-3) determines the choice between apoptotic or necrotic cell death in Cd+ 2-exposed human proximal tubule cells. Toxicological Sciences. 2004;80: 358–366. doi: 10.1093/toxsci/kfh158 15129022

[20] Somji S, Garrett SH, Sens MA, Sens DA. The unique N-terminal sequence of metallothionein-3 is required to regulate the choice between apoptotic or necrotic cell death of human proximal tubule cells exposed to Cd+ 2. Toxicological Sciences. 2006;90: 369–376. doi: 10.1093/toxsci/kfj089 16387743

[21] Kim D, Garrett SH, Sens MA, Somji S, Sens DA. Metallothionein isoform 3 and proximal tubule vectorial active transport. Kidney international. 2002;61: 464–472. doi: 10.1046/j.1523-1755.2002.00153.x 11849386

[22] Bathula CS, Garrett SH, Zhou XD, Sens MA, Sens DA, Somji S. Cadmium, vectorial active transport, and MT-3—dependent regulation of cadherin expression in human proximal tubular cells. Toxicological Sciences. 2008;102: 310–318. doi: 10.1093/toxsci/kfn004 18182399

[23] Slusser A, Bathula CS, Sens DA, Somji S, Sens MA, Zhou XD, et al. Cadherin expression, vectorial active transport, and metallothionein isoform 3 mediated EMT/MET responses in cultured primary and immortalized human proximal tubule cells. PLoS One. 2015;10: e0120132. doi: 10.1371/journal.pone.0120132 25803827 PMC4372585

[24] Garrett SH, Sens MA, Shukla D, Nestor S, Somji S, Todd JH, et al. Metallothionein isoform 3 expression in the human prostate and cancer-derived cell lines. The Prostate. 1999;41: 196–202. doi: 10.1002/(sici)1097-0045(19991101)41:3&lt;196::aid-pros7&gt;3.0.co;2-u 10517878

[25] Sens MA, Somji S, Garrett SH, Beall CL, Sens DA. Metallothionein isoform 3 overexpression is associated with breast cancers having a poor prognosis. The American journal of pathology. 2001;159: 21–26. doi: 10.1016/S0002-9440(10)61668-9 11438449 PMC1850423

[26] Sens MA, Somji S, Lamm DL, Garrett SH, Slovinsky F, Todd JH, et al. Metallothionein isoform 3 as a potential biomarker for human bladder cancer. Environmental health perspectives. 2000;108: 413–418. doi: 10.1289/ehp.00108413 10811567 PMC1638035

[27] Somji S, Garrett SH, Zhou XD, Zheng Y, Sens DA, Sens MA. Absence of metallothionein 3 expression in breast cancer is a rare but favorable marker that is under epigenetic control. Toxicological & Environ Chemistry. 2010;92: 1673–1695. doi: 10.1080/02772241003711274 21170156 PMC3002175

[28] Werynska B, Pula B, Muszczynska-Bernhard B, Gomulkiewicz A, Jethon A, Podhorska-Okolow M, et al. Expression of metallothionein-III in patients with non-small cell lung cancer. Anticancer research. 2013;33: 965–974. 23482768

[29] Gomulkiewicz A, Jablonska K, Pula B, Grzegrzolka J, Borska S, Podhorska-Okolow M, et al. Expression of metallothionein 3 in ductal breast cancer. International journal of oncology. 2016;49: 2487–2497. doi: 10.3892/ijo.2016.3759 27840910

[30] Deng D, El-Rifai W, Ji J, Zhu B, Trampont P, Li J, et al. Hypermethylation of metallothionein-3 CpG island in gastric carcinoma. Carcinogenesis. 2003;24: 25–29. doi: 10.1093/carcin/24.1.25 12538345

[31] Smith E, Drew PA, Tian Z-Q, De Young NJ, Liu J-F, Mayne GC, et al. Metallothionien 3 expression is frequently down-regulated in oesophageal squamous cell carcinoma by DNA methylation. Molecular Cancer. 2005;4: 1–9. doi: 10.1186/1476-4598-4-1 16351731 PMC1343579

[32] Peng D, Hu T-L, Jiang A, Washington MK, Moskaluk CA, Schneider-Stock R, et al. Location-specific epigenetic regulation of the metallothionein 3 gene in esophageal adenocarcinomas. PloS one. 2011;6: e22009. doi: 10.1371/journal.pone.0022009 21818286 PMC3139601

[33] El Ghazi I, Martin BL, Armitage IM. New proteins found interacting with brain metallothionein-3 are linked to secretion. International Journal of Alzheimer’s Disease. 2011;2011. 10.4061/2011/208634 PMC3014675 21234102

[34] Larson J, Yasmin T, Sens DA, Zhou XD, Sens MA, Garrett SH, et al. SPARC gene expression is repressed in human urothelial cells (UROtsa) exposed to or malignantly transformed by cadmium or arsenite. Toxicology Letters. 2010;199: 166–172. doi: 10.1016/j.toxlet.2010.08.020 20837119 PMC2956785

[35] Zhang R, Wang L, Garrett SH, Sens DA, Dunlevy JR, Zhou XD, et al. Elevated connexin 43 expression in arsenite-and cadmium-transformed human bladder cancer cells, tumor transplants and selected high grade human bladder cancers. Experimental and Toxicologic Pathology. 2016;68: 479–491. doi: 10.1016/j.etp.2016.08.003 27531258 PMC5123665

[36] Webb B, Sali A. Comparative protein structure modeling using MODELLER. Current protocols in bioinformatics. 2014;47: 5–6. doi: 10.1002/0471250953.bi0506s47 25199792

[37] Fiser A, Sali A. ModLoop: automated modeling of loops in protein structures. Bioinformatics. 2003;19: 2500–2501. doi: 10.1093/bioinformatics/btg362 14668246

[38] Jumper J, Evans R, Pritzel A, Green T, Figurnov M, Ronneberger O, et al. Highly accurate protein structure prediction with AlphaFold. Nature. 2021;596: 583–589. doi: 10.1038/s41586-021-03819-2 34265844 PMC8371605

[39] Laskowski RA, MacArthur MW, Thornton JM. PROCHECK: validation of protein-structure coordinates. 2006.

[40] Laskowski RA, Jabłońska J, Pravda L, Va\vreková RS, Thornton JM. PDBsum: Structural summaries of PDB entries. Protein science. 2018;27: 129–134. doi: 10.1002/pro.3289 28875543 PMC5734310

[41] Schrödinger LLC. The {PyMOL} Molecular Graphics System, Version~1.8. 2015 Nov.

[42] Morris GM, Huey R, Lindstrom W, Sanner MF, Belew RK, Goodsell DS, et al. AutoDock4 and AutoDockTools4: Automated docking with selective receptor flexibility. Journal of computational chemistry. 2009;30: 2785–2791. doi: 10.1002/jcc.21256 19399780 PMC2760638

[43] Weng G, Wang E, Wang Z, Liu H, Zhu F, Li D, et al. HawkDock: a web server to predict and analyze the protein—protein complex based on computational docking and MM/GBSA. Nucleic acids research. 2019;47: W322—W330. doi: 10.1093/nar/gkz397 31106357 PMC6602443

[44] Hetényi C, Spoel D van der. Toward prediction of functional protein pockets using blind docking and pocket search algorithms. Protein Science. 2011;20: 880–893. doi: 10.1002/pro.618 21413095 PMC3125872

[45] Genheden S, Ryde U. The MM/PBSA and MM/GBSA methods to estimate ligand-binding affinities. Expert opinion on drug discovery. 2015;10: 449–461. doi: 10.1517/17460441.2015.1032936 25835573 PMC4487606

[46] Hou T, Wang J, Li Y, Wang W. Assessing the performance of the molecular mechanics/Poisson Boltzmann surface area and molecular mechanics/generalized Born surface area methods. II. The accuracy of ranking poses generated from docking. Journal of computational chemistry. 2011;32: 866–877. doi: 10.1002/jcc.21666 20949517 PMC3043139

[47] Xue LC, Rodrigues JP, Kastritis PL, Bonvin AM, Vangone A. PRODIGY: a web server for predicting the binding affinity of protein—protein complexes. Bioinformatics. 2016;32: 3676–3678. doi: 10.1093/bioinformatics/btw514 27503228

[48] Dominguez R. A common binding site for actin-binding proteins on the actin surface. Actin-Monomer-Binding Proteins. Springer; 2007.

[49] Detrisac CJ, Sens MA, Garvin AJ, Spicer SS, Sens DA. Tissue culture of human kidney epithelial cells of proximal tubule origin. Kidney international. 1984;25: 383–390. doi: 10.1038/ki.1984.28 6727133

[50] Ryan MJ, Johnson G, Kirk J, Fuerstenberg SM, Zager RA, Torok-Storb B. HK-2: an immortalized proximal tubule epithelial cell line from normal adult human kidney. Kidney international. 1994;45: 48–57. doi: 10.1038/ki.1994.6 8127021

[51] Lever JE. Inducers of dome formation in epithelial cell cultures including agents that cause differentiation. Tissue culture of epithelial cells. Springer; 1985. pp. 3–22.

[52] Sens DA, Detrisac CJ, Sens MA, Rossi MR, Wenger SL, Todd JH. Tissue culture of human renal epithelial cells using a defined serum-free growth formulation. Nephron Experimental Nephrology. 1999;7: 344–352. doi: 10.1159/000020632 10559632

[53] Risi CM, Pepper I, Belknap B, Landim-Vieira M, White HD, Dryden K, et al. The structure of the native cardiac thin filament at systolic Ca2+ levels. Proceedings of the National Academy of Sciences. 2021;118. doi: 10.1073/pnas.2024288118 33753506 PMC8020778

[54] Kusakabe T, Motoki K, Hori K. Mode of interactions of human aldolase isozymes with cytoskeletons. Archives of Biochemistry and Biophysics. 1997. doi: 10.1006/abbi.1997.0204 9244396

[55] Du S, Guan Z, Hao L, Song Y, Wang L, Gong L, et al. Fructose-bisphosphate aldolase a is a potential metastasis-associated marker of lung squamous cell carcinoma and promotes lung cell tumorigenesis and migration. PLoS ONE. 2014. doi: 10.1371/journal.pone.0085804 24465716 PMC3900443

[56] Knipp M, Meloni G, Roschitzki B, Vašák M. Zn7metallothionein-3 and the synaptic vesicle cycle: interaction of metallothionein-3 with the small GTPase Rab3A. Biochemistry. 2005;44: 3159–3165. doi: 10.1021/bi047636d 15736926

[57] Homma Y, Hiragi S, Fukuda M. Rab family of small GTPases: an updated view on their regulation and functions. The FEBS journal. 2021;288: 36–55. doi: 10.1111/febs.15453 32542850 PMC7818423

[58] Christensen EI, Verroust PJ, Nielsen R. Receptor-mediated endocytosis in renal proximal tubule. Pflügers Archiv-European Journal of Physiology. 2009;458: 1039–1048. doi: 10.1007/s00424-009-0685-8 19499243

[59] Lahti DW, Hoekman JD, Tokheim AM, Martin BL, Armitage IM. Identification of mouse brain proteins associated with isoform 3 of metallothionein. Protein science. 2005;14: 1151–1157. doi: 10.1110/ps.041113005 15802640 PMC2253260

[60] Lee S-J, Cho K-S, Kim HN, Kim H-J, Koh J-Y. Role of zinc metallothionein-3 (ZnMt3) in epidermal growth factor (EGF)-induced c-Abl protein activation and actin polymerization in cultured astrocytes. Journal of Biological Chemistry. 2011;286: 40847–40856. doi: 10.1074/jbc.M111.245993 21900236 PMC3220504

[61] Lee S-J, Seo B-R, Koh J-Y. Metallothionein-3 modulates the amyloid β endocytosis of astrocytes through its effects on actin polymerization. Molecular brain. 2015;8: 1–12. doi: 10.1186/s13041-014-0092-8 26637294 PMC4670512

[62] Cai B, Ding Z-C, Zhang Q, Ni F-Y, Wang H, Zheng Q, et al. The structural and biological significance of the EAAEAE insert in the α-domain of human neuronal growth inhibitory factor. The FEBS journal. 2009;276: 3547–3558. doi: 10.1111/j.1742-4658.2009.07075.x 19490120

[63] Ding Z-C, Zheng Q, Cai B, Yu W-H, Teng X-C, Wang Y, et al. Effect of α-domain substitution on the structure, property and function of human neuronal growth inhibitory factor. JBIC Journal of Biological Inorganic Chemistry. 2007;12: 1173–1179. doi: 10.1007/s00775-007-0287-x 17712581

[64] Ding Z-C, Ni F-Y, Huang Z-X. Neuronal growth-inhibitory factor (metallothionein-3): structure—function relationships. The FEBS journal. 2010;277: 2912–2920. doi: 10.1111/j.1742-4658.2010.07716.x 20561055

[65] Ni F-Y, Cai B, Ding Z-C, Zheng F, Zhang M-J, Wu H-M, et al. Structural prediction of the β-domain of metallothionein-3 by molecular dynamics simulation. Proteins: Structure, Function, and Bioinformatics. 2007;68: 255–266. 10.1002/prot.21404 17427961

[66] Dominguez R. Actin-binding proteins—A unifying hypothesis. Trends in Biochemical Sciences. 2004. doi: 10.1016/j.tibs.2004.09.004 15501675

[67] Pollard TD. Actin and actin-binding proteins. Cold Spring Harbor Perspectives in Biology. 2016. doi: 10.1101/cshperspect.a018226 26988969 PMC4968159

[68] Voels B, Wang L, Sens DA, Garrett SH, Zhang K, Somji S. The unique C-and N-terminal sequences of Metallothionein isoform 3 mediate growth inhibition and Vectorial active transport in MCF-7 cells. BMC cancer. 2017;17: 1–13. doi: 10.1186/s12885-016-3022-6 28545470 PMC5445401

[69] Ullah MdA, Sarkar B, Islam SS. Exploiting the reverse vaccinology approach to design novel subunit vaccines against Ebola virus. Immunobiology. 2020;225: 151949. doi: 10.1016/j.imbio.2020.151949 32444135

[70] Humayun F, Khan A, Ahmad S, Yuchen W, Wei G, Nizam-Uddin N, et al. Abrogation of SARS-CoV-2 interaction with host (NRP1) neuropilin-1 receptor through high-affinity marine natural compounds to curtail the infectivity: A structural-dynamics data. Computers in Biology and Medicine. 2022;141: 104714. doi: 10.1016/j.compbiomed.2021.104714 34772509 PMC8324387

[71] Zhang X, Perez-Sanchez H, C. Lightstone F. A Comprehensive Docking and MM/GBSA Rescoring Study of Ligand Recognition upon Binding Antithrombin. Current Topics in Medicinal Chemistry. 2017;17: 1631–1639. doi: 10.2174/1568026616666161117112604 27852201 PMC5403970

[72] Akachar J, Bouricha EM, Hakmi M, Belyamani L, el Jaoudi R, Ibrahimi A. Identifying epitopes for cluster of differentiation and design of new peptides inhibitors against human SARS-CoV-2 spike RBD by an in-silico approach. Heliyon. 2020;6: e05739. doi: 10.1016/j.heliyon.2020.e05739 33364503 PMC7753134

[73] Jang S, Nelson JC, Bend EG, Rodr\’\iguez-Laureano L, Tueros FG, Cartagenova L, et al. Glycolytic enzymes localize to synapses under energy stress to support synaptic function. Neuron. 2016;90: 278–291. doi: 10.1016/j.neuron.2016.03.011 27068791 PMC4840048

[74] Jella KK, Yu L, Yue Q, Friedman D, Duke BJ, Alli AA. Exosomal GAPDH from proximal tubule cells regulate ENaC activity. PloS one. 2016;11: e0165763. doi: 10.1371/journal.pone.0165763 27802315 PMC5089749

[75] Qiao G, Wu A, Chen X, Tian Y, Lin X. Enolase 1, a Moonlighting Protein, as a Potential Target for Cancer Treatment. International journal of biological sciences. 2021;17: 3981. doi: 10.7150/ijbs.63556 34671213 PMC8495383

[76] Hui MH, Rhine K, Tolan DR. Actin filament-and Wiskott-Aldrich syndrome protein-binding sites on fructose-1, 6-bisphosphate aldolase are functionally distinct from the active site. Cytoskeleton. 2021;78: 129–141. doi: 10.1002/cm.21646 33210455

[77] Koh J-Y, Lee S-J. Metallothionein-3 as a multifunctional player in the control of cellular processes and diseases. Molecular Brain. 2020;13: 1–12. doi: 10.1186/s13041-019-0541-5 32843100 PMC7448430

[78] Vašák M, Meloni G. Mammalian metallothionein-3: New functional and structural insights. International journal of molecular sciences. 2017;18: 1117. doi: 10.3390/ijms18061117 28538697 PMC5485941

[79] Vignesh KS, Figueroa JAL, Porollo A, Divanovic S, Caruso JA, Deepe GS Jr. IL-4 induces metallothionein 3-and SLC30A4-dependent increase in intracellular Zn2+ that promotes pathogen persistence in macrophages. Cell reports. 2016;16: 3232–3246. doi: 10.1016/j.celrep.2016.08.057 27653687 PMC5603080

[80] Suzuki M, Ramezanpour M, Cooksley C, Ogi K, Psaltis AJ, Nakamaru Y, et al. Metallothionein-3 is a clinical biomarker for tissue zinc levels in nasal mucosa. Auris Nasus Larynx. 2021. doi: 10.1016/j.anl.2021.01.019 33526321

[81] Garrett SH, Phillips V, Somji S, Sens MA, Dutta R, Park S, et al. Transient induction of metallothionein isoform 3 (MT-3), c-fos, c-jun and c-myc in human proximal tubule cells exposed to cadmium. Toxicology letters. 2002;126: 69–80. doi: 10.1016/s0378-4274(01)00448-9 11738272

[82] Roohani N, Hurrell R, Kelishadi R, Schulin R. Zinc and its importance for human health: An integrative review. Journal of research in medical sciences: the official journal of Isfahan University of Medical Sciences. 2013;18: 144. 23914218 PMC3724376

[83] Miyoshi Y, Tanabe S, Suzuki T. Cellular zinc is required for intestinal epithelial barrier maintenance via the regulation of claudin-3 and occludin expression. American Journal of Physiology-Gastrointestinal and Liver Physiology. 2016;311: G105—G116. doi: 10.1152/ajpgi.00405.2015 27151944

[84] Li F, Abuarab N, Sivaprasadarao A. Reciprocal regulation of actin cytoskeleton remodelling and cell migration by Ca2+ and Zn2+: role of TRPM2 channels. Journal of cell science. 2016;129: 2016–2029. doi: 10.1242/jcs.179796 27068538

