# Supplementary material for: Protein interactions with metallothionein-3 promote vectorial active transport in human proximal tubular cells
Source: PLoS One. 2022 May 3;17(5):e0267599. doi: 10.1371/journal.pone.0267599 (PMC9064079; doi:10.1371/journal.pone.0267599)
Supplement: S1 Table — (DOCX) [file pone.0267599.s001.docx]

| **S1 Table Primary antibodies.** | | | | |
| --- | --- | --- | --- | --- |
| **Antibody** | **WB Dilution** | **Immunolocalization** | **Company** | **Catalog #** |
| β-actin | 1:1000 | NA | Abcam | Ab8226 |
| Tropomyosin a1 | 1:1000 | 1:20 | Santa-Cruz | Sc47199 |
| Myosin -9 | 1:500 | 1:20 | Abcam | Ab55456 |
| Enolase-1 | 1:3000 | 1:1000 | Abcam | Ab56795 |
| aldolase-a | 1:500 | 1:20 | Santa-Cruz | Sc12059 |
| V5 | 1:5000 | NA | Invitrogen | Ab9116 |
| MT-3 | 1:100 | 1:50 | [13] | [13] |
| Phalloidin (F-actin) | NA | 1:100 | Life Technologies | A12380 |
