# Supplementary material for: Protein interactions with metallothionein-3 promote vectorial active transport in human proximal tubular cells
Source: PLoS One. 2022 May 3;17(5):e0267599. doi: 10.1371/journal.pone.0267599 (PMC9064079; doi:10.1371/journal.pone.0267599)
Supplement: S1 Raw images — PDF file containing TIFF images of all raw uncropped Western blot results. A. Blots used to generate Fig 1A. B and C. Blots used to generate Fig 1B. X indicates lanes not used. (PDF) [file pone.0267599.s002.pdf]

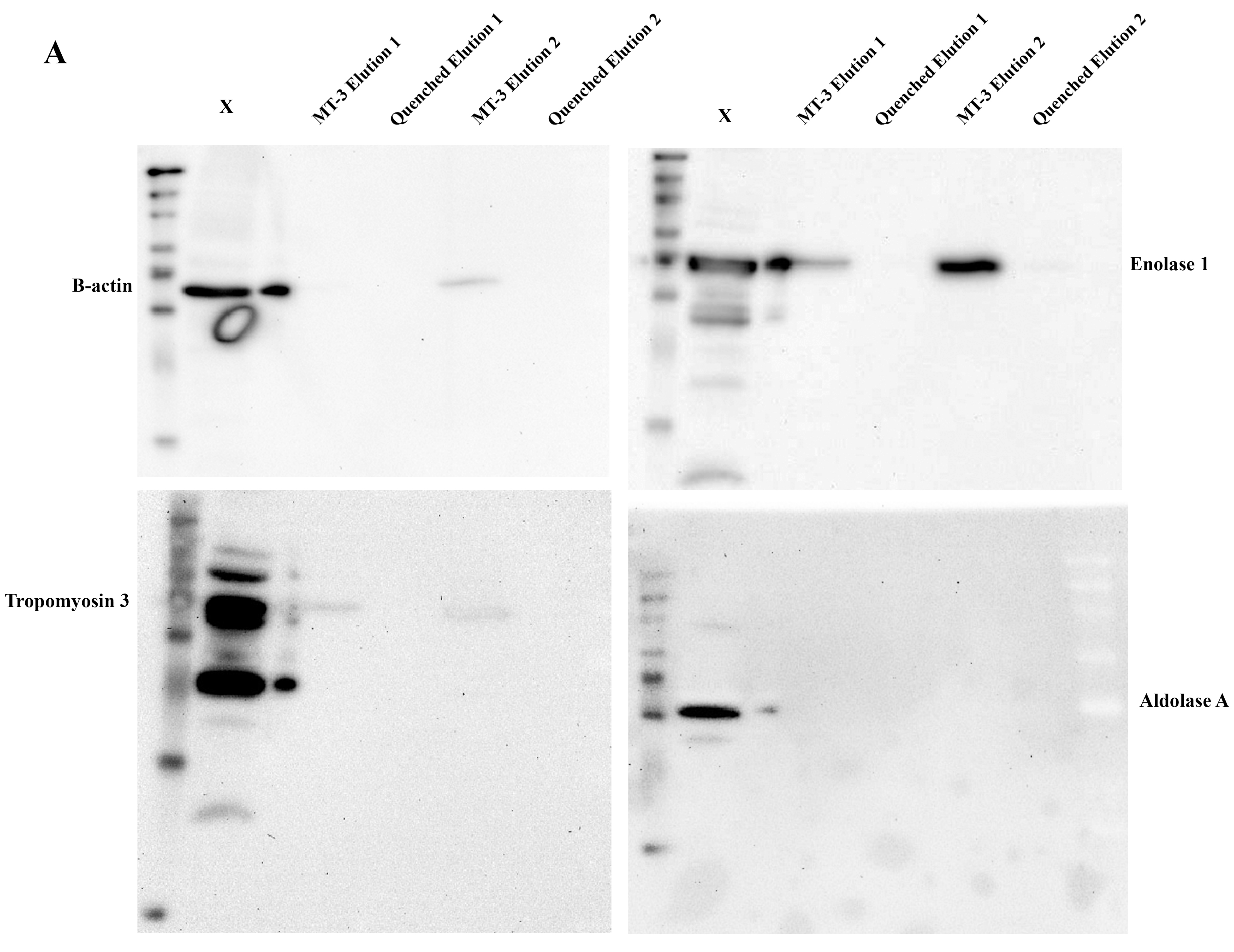
A
X
MT-3 Elution 1
Quenched Elution 1
MT-3 Elution 2
Quenched Elution 2
X
MT-3 Elution 1
Quenched Elution 1
MT-3 Elution 2
Quenched Elution 2
B-actin
Enolase 1
Tropomyosin 3
Aldolase A

B

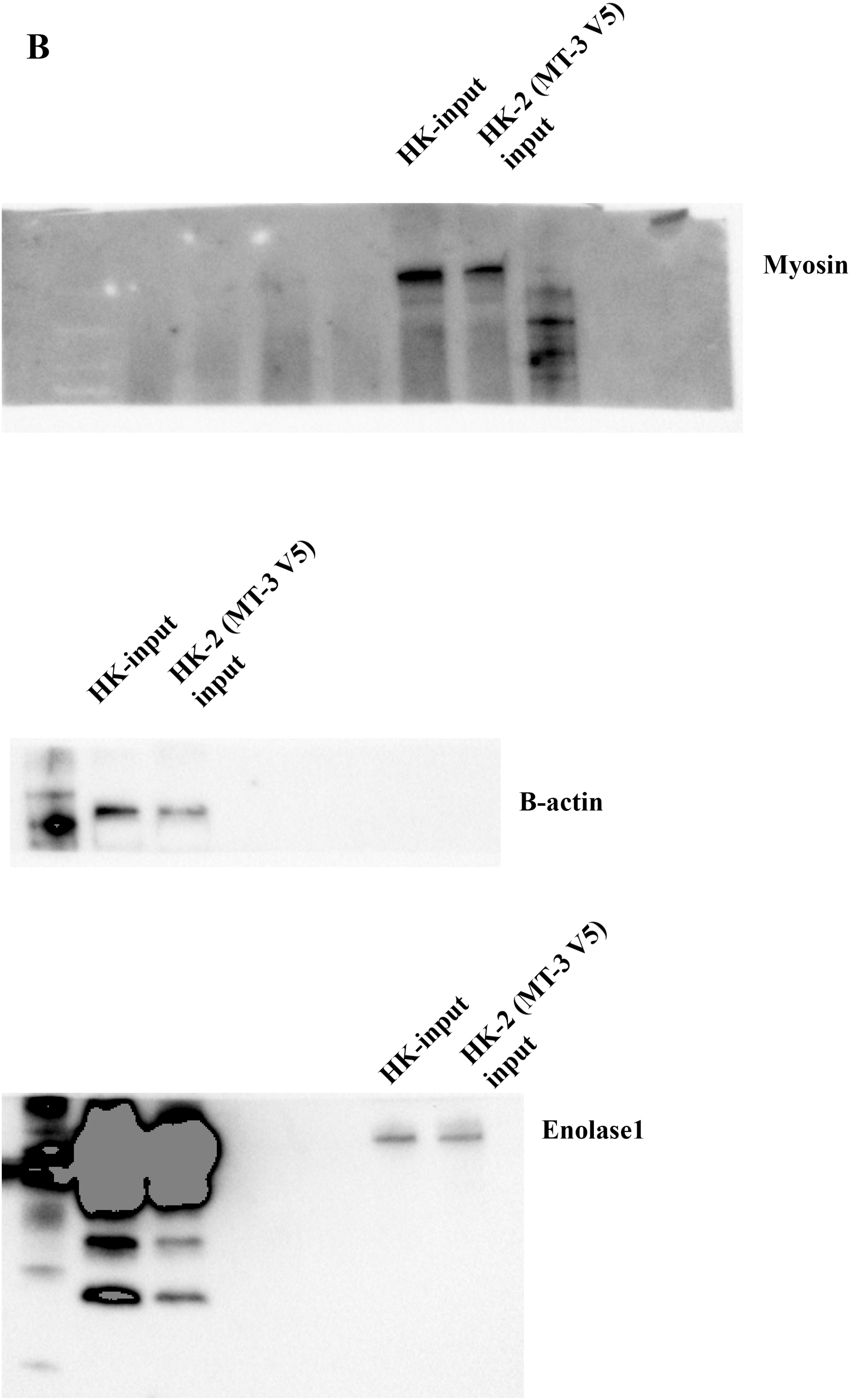
HK-input
HK-2 (MT-3 V5) input
Myosin
HK-input
HK-2 (MT-3 V5) input
B-actin
HK-input
HK-2 (MT-3 V5) input
Enolase1

C

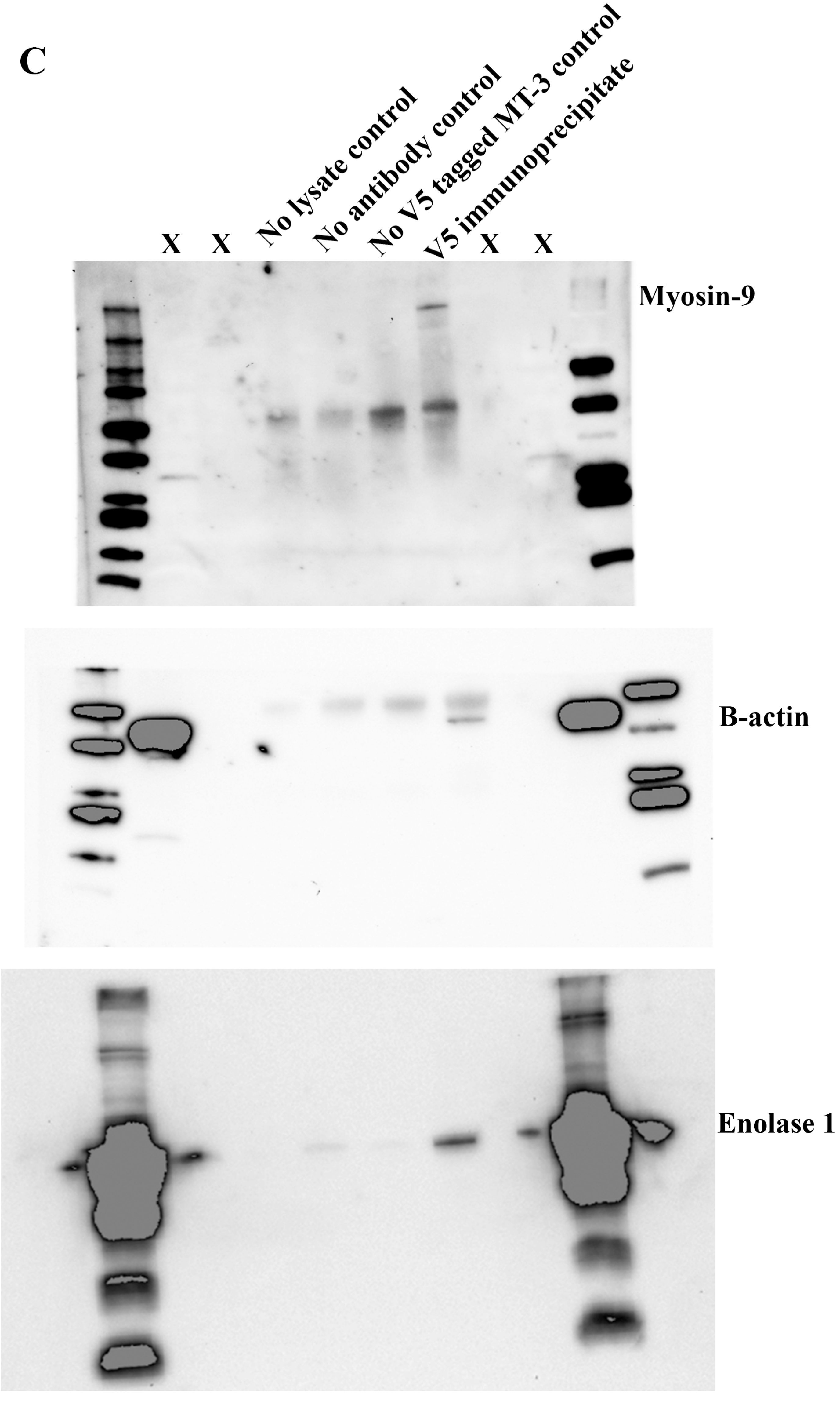
X
X
No lysate control
No antibody control
No V5 tagged MT-3 control
V5 immunoprecipitate
X
X
Myosin-9
B-actin
Enolase 1
