# Supplementary figures and images for: Protein interactions with metallothionein-3 promote vectorial active transport in human proximal tubular cells
Source: PLoS One. 2022 May 3;17(5):e0267599. doi: 10.1371/journal.pone.0267599 (PMC9064079; doi:10.1371/journal.pone.0267599)

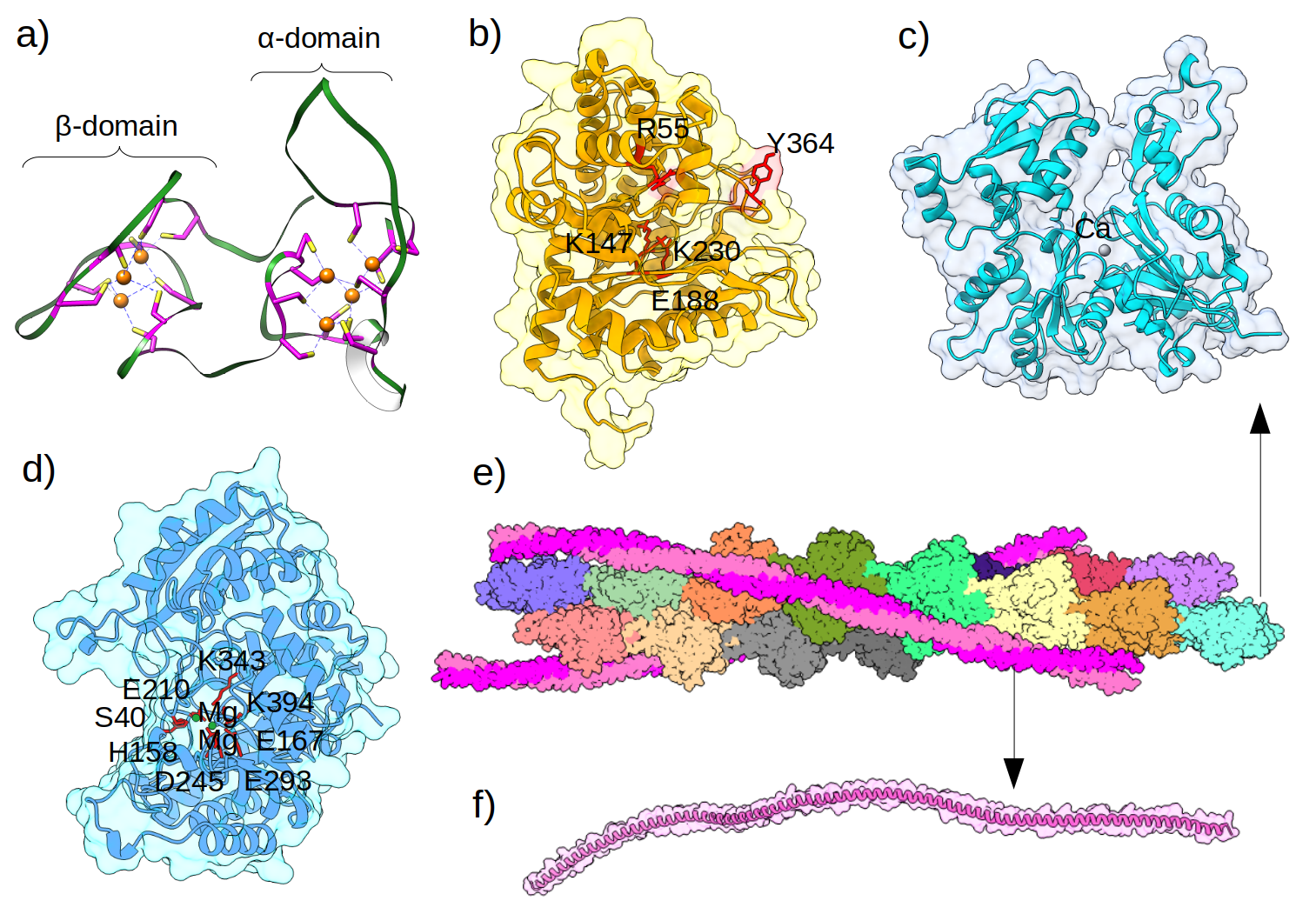

Supplement: S1 Fig — a). MT-3 and it’s two domains with β- and α-domain linked at K31-K32, the metal atoms (Zn2+) shown as orange spheres are encapsulated with Cys shown as pink sticks, sulfur shown in yellow, and each Zn2+ is linked with four other atoms forming a tetrahedral geometry. b). Aldolase A with active site residues highlighted in red and labeled. The primary catalytic residues are K229, E187, and K147 and the secondary site residues are R55 and Y363. c). β-actin in its monomeric form containing a Ca2+ atom. d) Enolase 1 with active site residues highlighted in red and labeled. The metal binding residues are S40, D245, E293, D318 and the substrate binding site residues include H158, E167, E210, K343, and K394. e). The oligomeric form of β-actin and tropomyosin 3 complex. The actin molecules are shown in the middle of the filament colored by single monomeric entities and tropomyosin 3 is shown in its homo-dimer form on each side of the filament colored in pink. f). Tropomyosin 3 as a single entity. (TIF) [file pone.0267599.s003.tif]

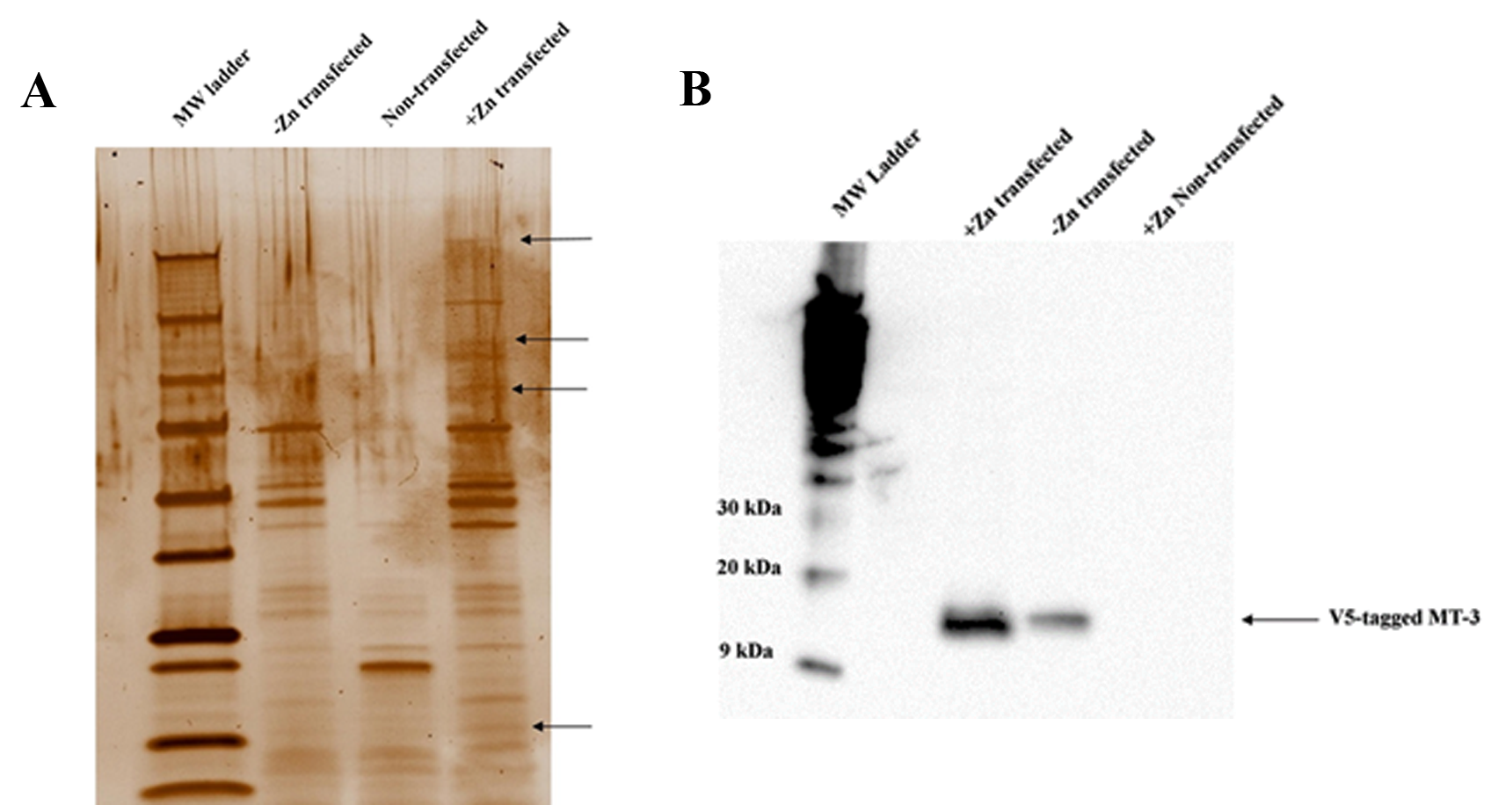

Supplement: S2 Fig — Co-immunoprecipitation of V5-tagged MT-3 and putative protein interactants. Eluates were subjected to SDS-PAGE and silver-staining (A) or western blotting (B) for MT-3. (TIF) [file pone.0267599.s004.tif]

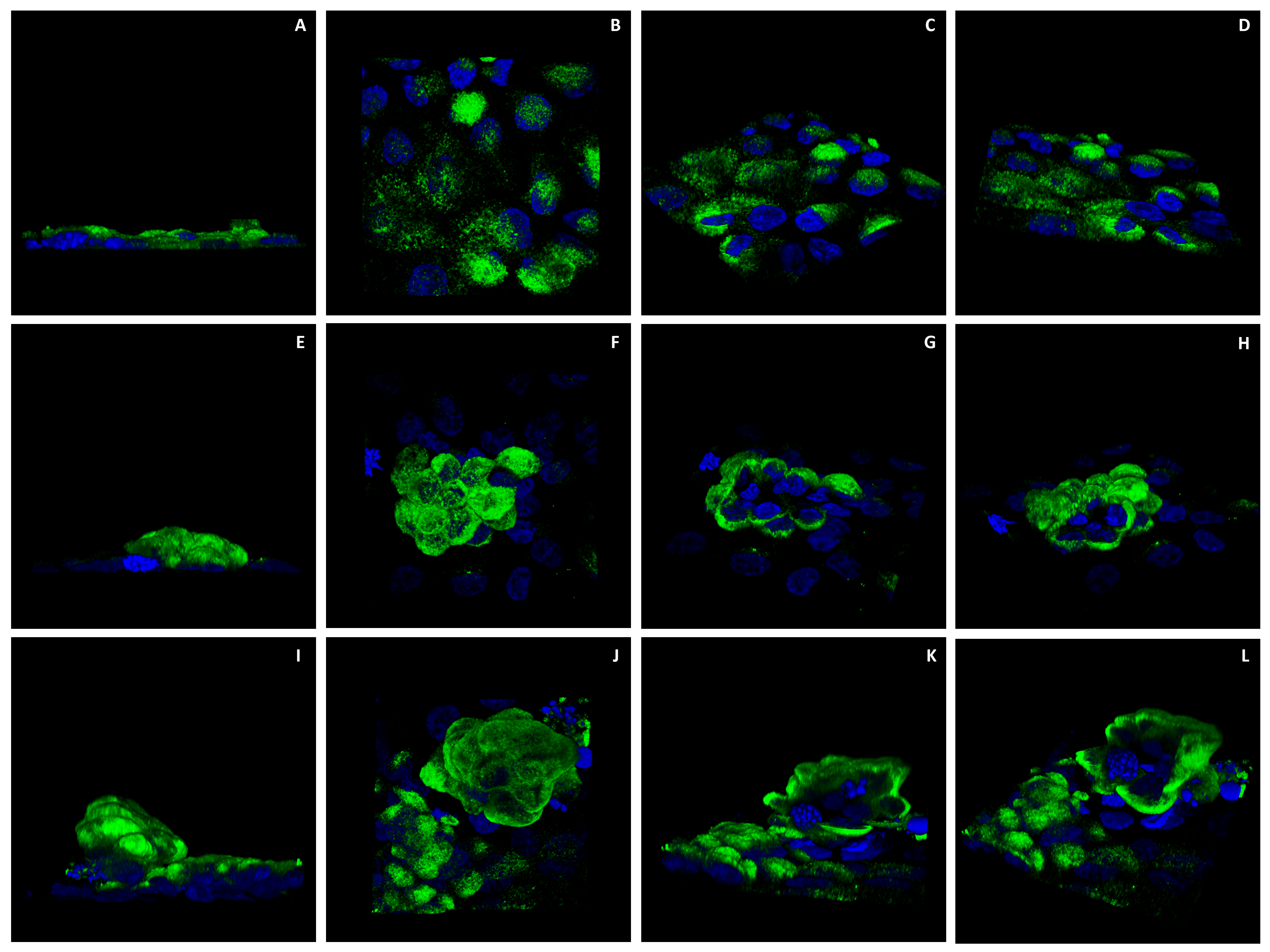

Supplement: S3 Fig — MT-3 (green) and nuclei (blue) are seen in 4 different 3D views. The fields are the same ones shown in Figs 1 and 2. The side angle view is shown in (A, E, I); a view of the apical surface from the top-down is shown in (B, F, J); two different angle views with a portion of the field cut away in a 3D slice (cut in the X, Y, and Z planes) is shown in (C-D, G-H, K-L). The non-doming monolayer (A-D), the moderately tall dome (E-H), and the taller dome structure (I-L) are all shown. (TIF) [file pone.0267599.s005.tif]

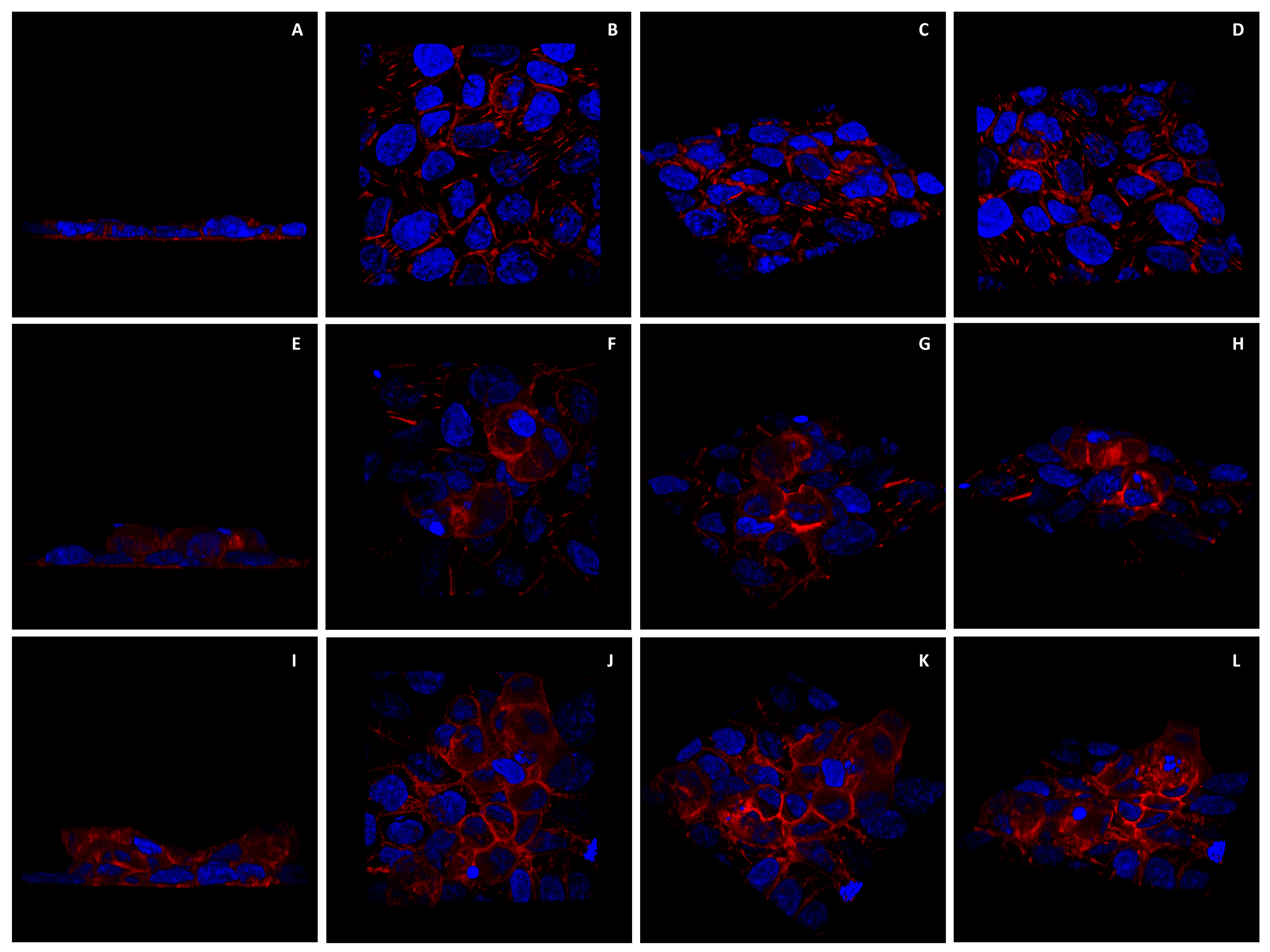

Supplement: S4 Fig — Three dimensional projection of F-actin intracellular localization. F-actin (red) and nuclei (blue) are seen in 4 different 3D views. The fields are the same ones shown in Figs 1 and 2. The side angle view is shown in (A, E, I); a view of the apical surface from the a top-down is shown in (B, F, J); two different angle views with a portion of the field cut away in a 3D slice (cut in the X, Y, and Z planes) is shown in (C-D, G-H, K-L). The non-doming monolayer (A-D), the moderately tall dome (E-H) and the taller dome structure (I-L) are all shown. (TIF) [file pone.0267599.s006.tif]

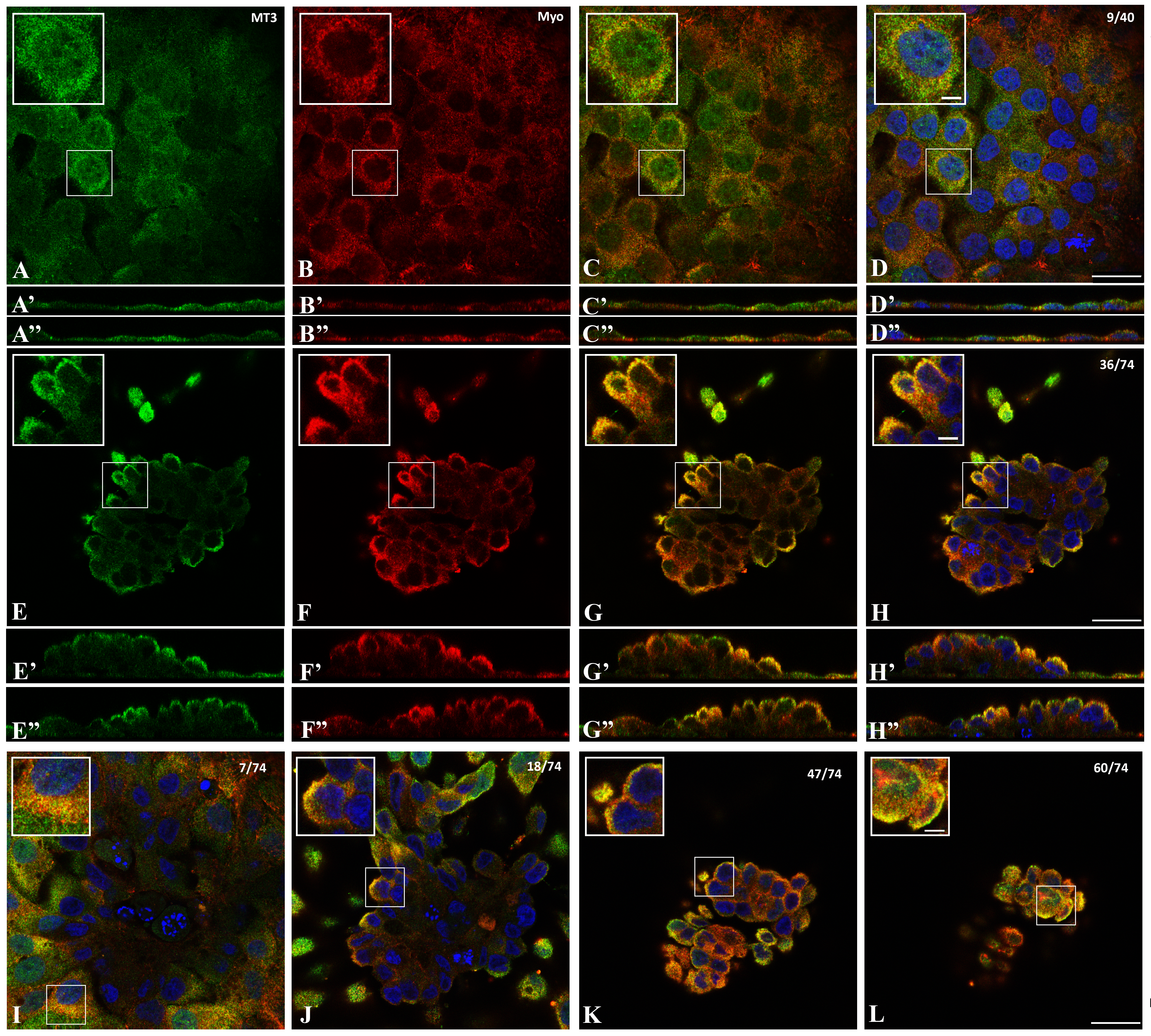

Supplement: S5 Fig — MT-3 (green, A and E) and myosin-9 (red, B and F) localization in HK-2 MT-3 cells. Respective orthogonal views are shown for each image and are indicated (’) for scanning along the x-axis and (") for scanning along the Y-axis. Panels C and G show co-localization (yellow) of MT-3 and myosin-9; panels D and H display the same co-localization with the addition of DAPI (blue) for nuclear visualization. Multiple regions of co-localization were found and are shown (I-L). (TIF) [file pone.0267599.s007.tif]

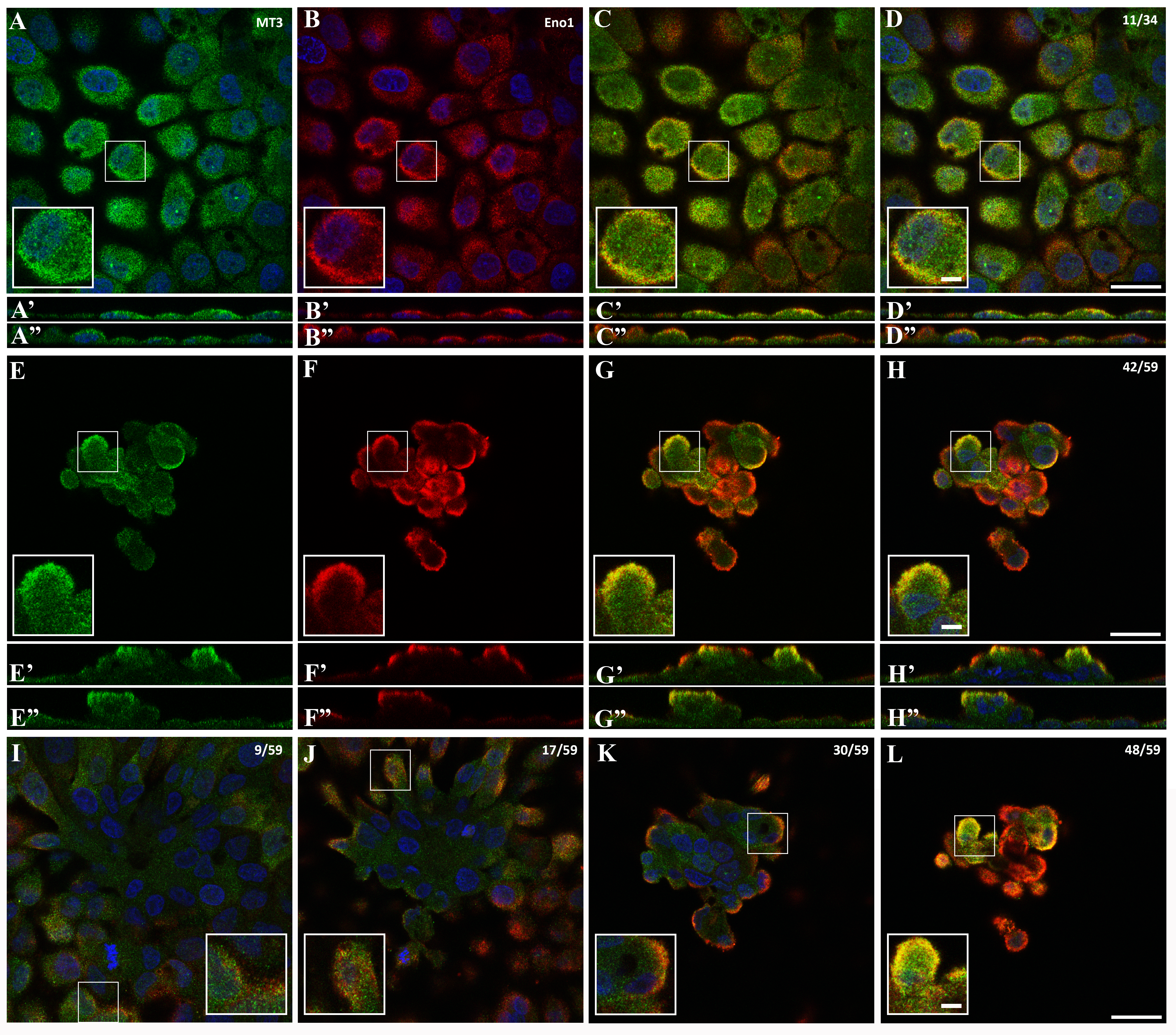

Supplement: S6 Fig — MT-3 (green, A and E) and enolase 1 (red, B and F) localization in HK-2 MT-3 cells. Respective orthogonal views are shown for each image and are indicated (’) for scanning along the x-axis and (") for scanning along the Y-axis. Panels C and G show co-localization (yellow) of MT-3 and enolase 1; panels D and H display the same co-localization with the addition of DAPI (blue) for nuclear visualization. Multiple regions of co-localization were found and are shown (I-L). (TIF) [file pone.0267599.s008.tif]

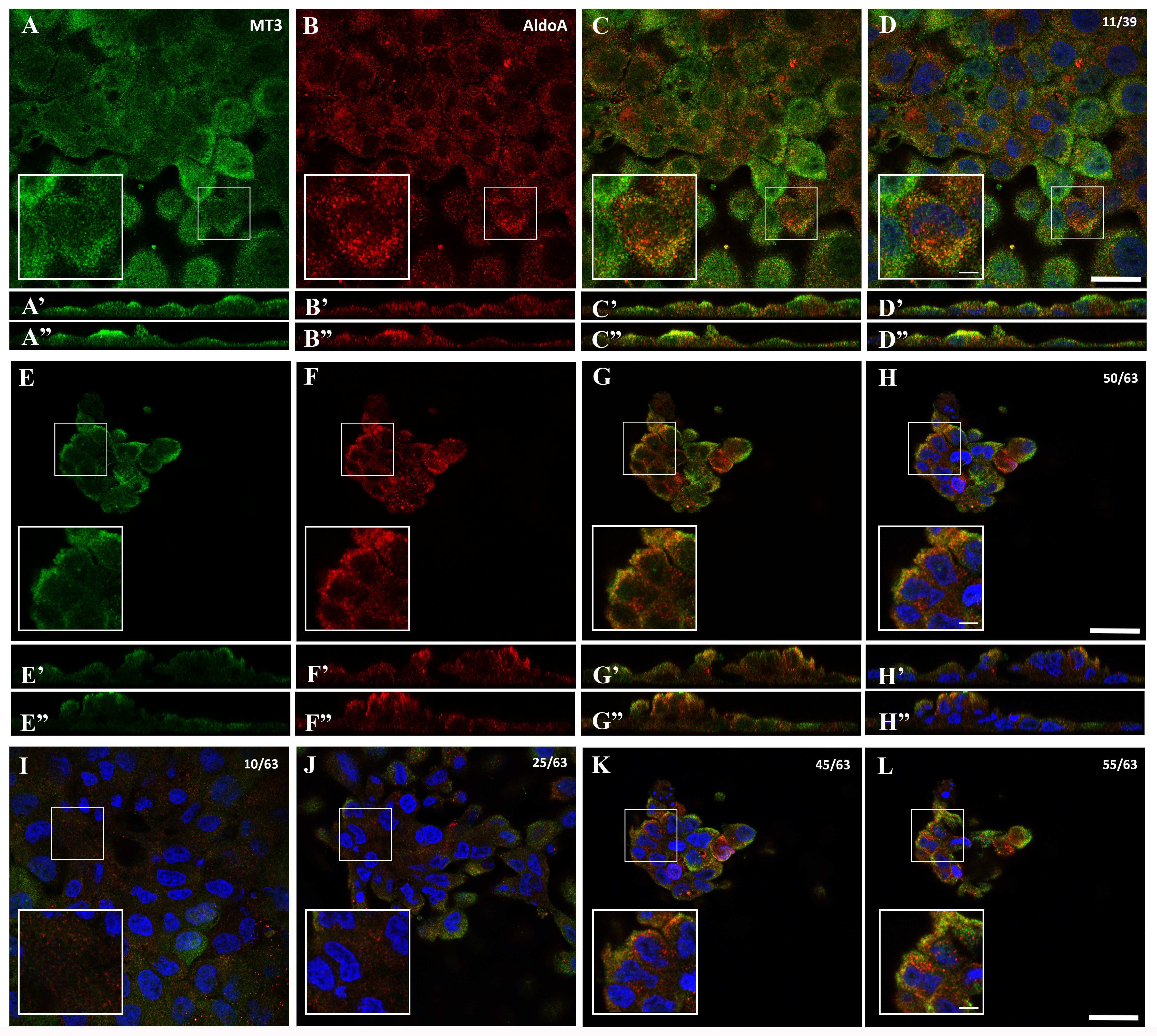

Supplement: S7 Fig — MT-3 (green, A and E) and aldolase A (red, B and F) localization in HK-2 MT-3 cells. Respective orthogonal views are shown for each image and are indicated (’) for scanning along the x-axis and (") for scanning along the Y-axis. Panels C and G show co-localization (yellow) of MT-3 and aldolase A; panels D and H display the same co-localization with the addition of DAPI (blue) for nuclear visualization. Multiple regions of co-localization were found and are shown (I-L). (TIF) [file pone.0267599.s009.tif]

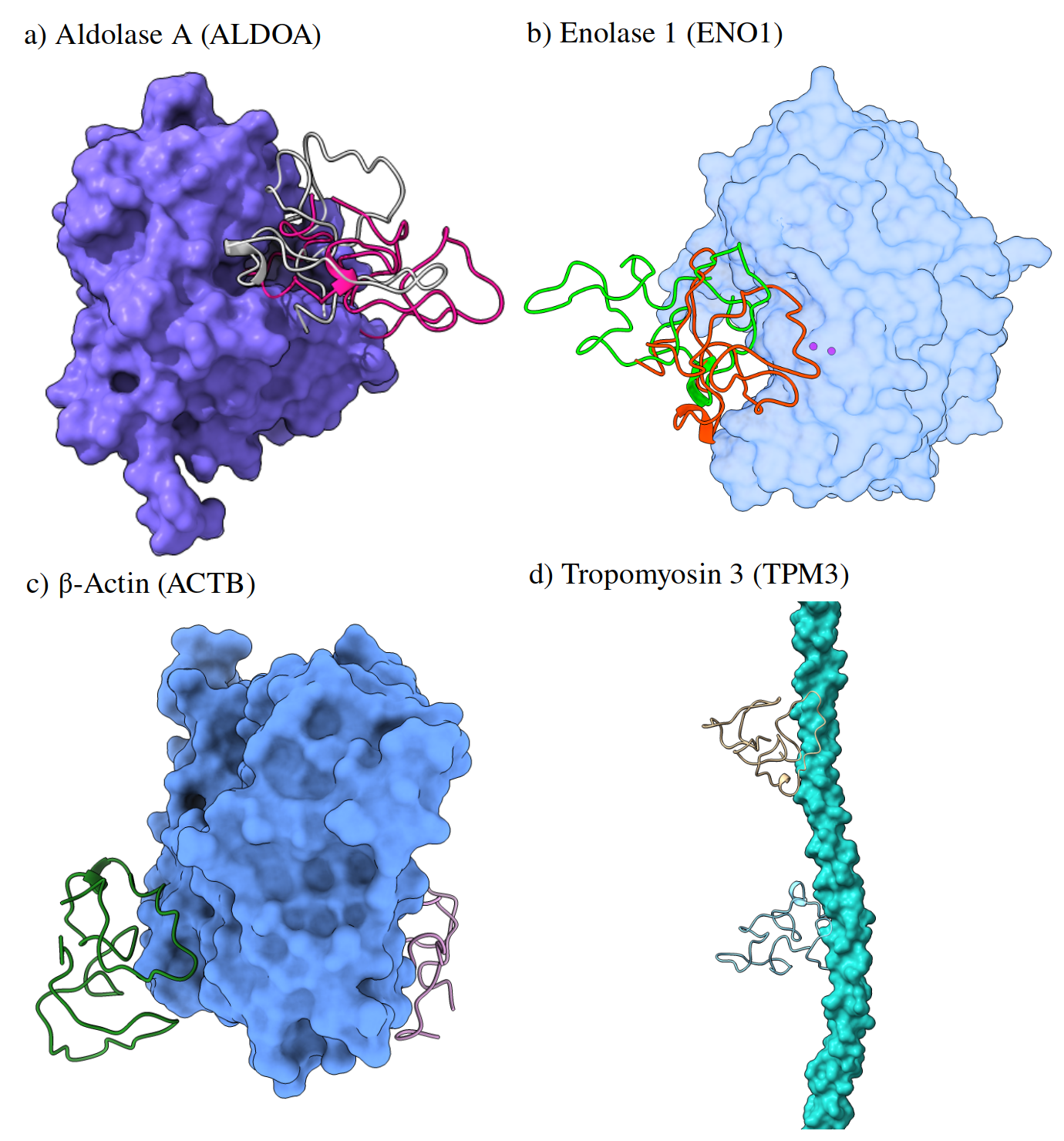

Supplement: S8 Fig — The figure shows two best poses of bound MT-3. a). Aldolase has binding affinities of -19.95 kcal/mol for grey conformation and -18.12 kcal/mol for magenta conformation of MT-3. It is seen that the best conformations are for poses that were around the active site. b). Enolase 1 has binding affinities of -7.67 kcal/mol for orange conformation and -7.25 kcal/mol for green conformation of MT-3. It is seen that best binding affinities are for poses that are bound near the catalytic site of the enzyme. c). β-actin has binding affinities of -7.54 kcal/mol for green conformation and -7.44 kcal/mol for pink conformation of MT-3. d). Tropomyosin 3 has binding affinities of -25.72 kcal/mol for purple conformation and -14.41 kcal/mol for blue conformation of MT-3. It is seen that many docked conformations clustered on these two positions. (TIF) [file pone.0267599.s010.tif]

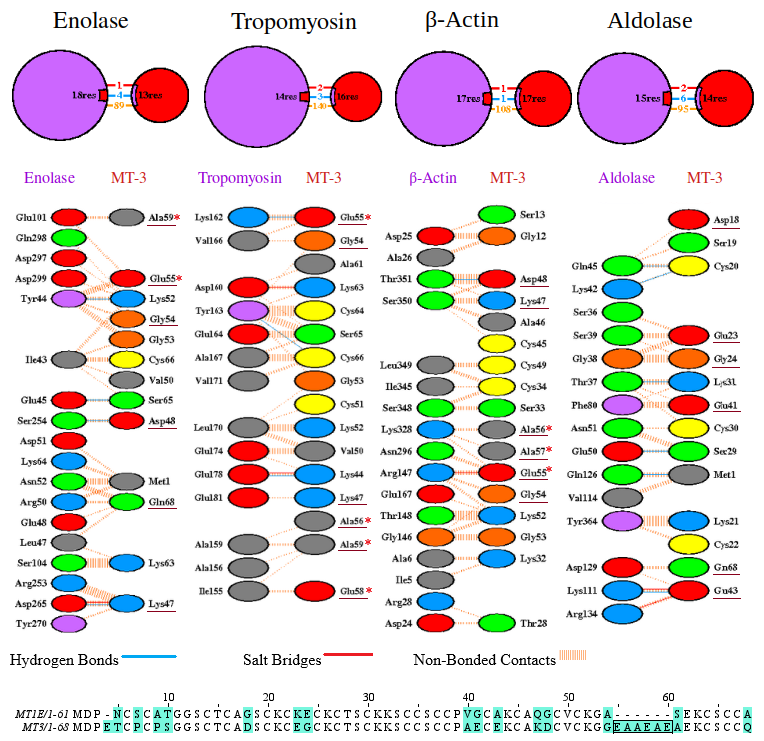

Supplement: S9 Fig — The number of interactions are given on the top with smaller circle representing MT-3 and larger being the binding partner. The interaction type is labeled and further it is seen that most interactions are with the α-domain of MT-3. The residues that are only present in MT-3 are underlined and the insert loop residues (EAAEAE) are marked with an asterisk (*). A pair-wise alignment of MT-3 and MT-1E is also shown to highlight these amino acids. (TIF) [file pone.0267599.s011.tif]

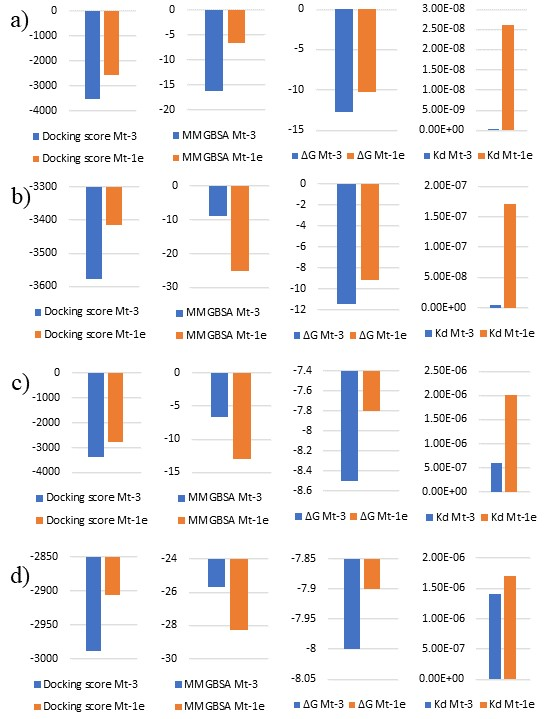

Supplement: S10 Fig — The protein-protein complexes of the binding partners of MT-3 were compared with docking models of MT-1E using parameters of docking score, MMGBSA affinity, ΔG, and Kd. a) Aldolase A showed stronger binding with MT-3 as compared to MT-1E in all the parameters; b) Enolase 1, c) β-actin and d) Tropomyosin showed stronger binding of MT-3 with its binding partners as compared to MT-1E on account of docking score, ΔG, and Kd. (TIF) [file pone.0267599.s012.tif]
